# Mechanosynthesis of Higher‐Order Cocrystals: Tuning Order, Functionality and Size in Cocrystal Design**

**DOI:** 10.1002/anie.202101248

**Published:** 2021-07-01

**Authors:** Zi Xuan Ng, Davin Tan, Wei Liang Teo, Felix León, Xiaoyan Shi, Ying Sim, Yongxin Li, Rakesh Ganguly, Yanli Zhao, Sharmarke Mohamed, Felipe García

**Affiliations:** ^1^ School of Physical and Mathematical Sciences Division of Chemistry and Biological Chemistry Nanyang Technological University 21 Nanyang Link 637371 Singapore Singapore; ^2^ School of Materials and Energy Guangdong University of Technology Guangzhou 510006 Guangdong P. R. China; ^3^ Department of Chemistry Shiv Nadar University NH91, Tehsil Dadri, Gautam Buddha Nagard 201314 Uttar Pradesh India; ^4^ Department of Chemistry Green Chemistry & Materials Modelling Laboratory Khalifa University of Science and Technology P.O. Box 127788 Abu Dhabi United Arab Emirates

**Keywords:** density functional theory, higher-order Cocrystals, mechanochemistry, phosphazanes

## Abstract

The ability to rationally design and predictably construct crystalline solids has been the hallmark of crystal engineering research. To date, numerous examples of multicomponent crystals comprising organic molecules have been reported. However, the crystal engineering of cocrystals comprising both organic and inorganic chemical units is still poorly understood and mostly unexplored. Here, we report a new diverse set of higher‐order cocrystals (HOCs) based on the structurally versatile—yet largely unexplored—phosph(V/V)azane heterosynthon building block. The novel ternary and quaternary cocrystals reported are held together by synergistic and orthogonal intermolecular interactions. Notably, the HOCs can be readily obtained either via sequential or one‐pot mechanochemical methods. Computational modelling methods reveal that the HOCs are thermodynamically driven to form and that their mechanical properties strongly depend on the composition and intermolecular forces in the crystal, offering untapped potential for optimizing material properties.

## Graphical abstract

Mechanosynthesis gives ternary and quaternary hybrid organic‐inorganic cocrystals held together via synergistic intermolecular interactions. Notably, higher‐order ternary and quaternary cocrystals can be readily obtained either via serial synthetic routes from the individual components or via one‐pot approaches.

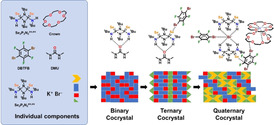

## Introduction

Cocrystals are multicomponent crystalline solids comprising at least two distinct chemical species in a periodic crystalline lattice.[^1^, ^2^, ^3^, ^4^] Their rational design and synthesis has been recognized as one of the most challenging aspects of crystal engineering. Whilst supramolecular synthons and energy frameworks have been successfully applied for the rational design of binary cocrystals, the toolkit for the construction of ternary and quaternary cocrystals (and beyond) is not well‐established.[^5^, ^6^, ^7^, ^8^, ^9^] We define a higher‐order cocrystal (HOC) as a solid‐form comprising more than two distinct chemical fragments that are sustained by a range of intermolecular forces. The rational discovery of HOCs provides the opportunity to widen the solid‐state properties of multiple active ingredients.

Recent advances in multicomponent cocrystallization strategies have been driven by the proven applications of these solid forms in pharmaceutical solids, as well as in functional materials (e.g., optical, photomechanical, inter alia).[^10^, ^11^, ^12^] However, most reports of multicomponent crystals relate to the synthesis of binary organic crystals such as salts or cocrystals.[^1^, ^2^] By contrast the development of cocrystals based on inorganic building blocks is still trailing behind their organic counterparts (vide infra). This is despite the fact that inorganic compounds have recently been proven to be good supramolecular building blocks. Braga[^13^, ^14^, ^15^, ^16^] and Zaworotko[^17^, ^18^, ^19^, ^20^, ^21^] introduced ionic cocrystals (ICCs) as a new class of highly tunable materials that not merely add, but synergistically combine the physicochemical properties of the individual components.^[22]^ However, with the exception of a handful of examples,[^23^, ^24^, ^25^, ^26^] their ICCs were limited to atomic salts as the inorganic building block.[^13^, ^14^, ^15^, ^16^, ^18^, ^27^, ^28^, ^29^] Such ICCs are a subset of the wider class of materials known as HOCs.

Recently, cyclodiphosphazane species[^30^, ^31^] have been demonstrated as robust^[32]^ and versatile[^33^, ^34^, ^35^] supramolecular building blocks in binary cocrystal formation.[^33^, ^36^, ^37^, ^38^, ^39^] However their use for the synthesis of multicomponent crystals beyond binary systems, remains unexplored.

Over the last decades, there has been an evolution of the strategies for the design and synthesis of higher‐order multicomponent cocrystals. Historically, it has been common to exploit different intermolecular interactions whilst considering the hierarchy, strength, and stabilization of the intermolecular interaction(s) present (***Strategy I***). Aakeroy et al. demonstrated that weaker amide⋅⋅⋅amide hydrogen bonds (HBs) were preferentially replaced with more thermodynamically favorable heteromeric acid⋅⋅⋅amide interactions to form ternary cocrystals.^[40]^ This strategy was further discussed by Mandal et al. and Nangia et al. who identified the hierarchy of the different intermolecular interactions of HBs, halogen bonds (HalB), and π‐π stacking through computational simulations,^[41]^ and used robust sulfonamide⋅⋅⋅pyridine—amides⋅⋅⋅lactam heterosynthons,^[6]^ respectively.

The second strategy is the use of isostructurality, and structural inequivalences between supramolecular interactions (***Strategy II***). Desiraju et al. employed supramolecular homologation to obtain organic ternary and quaternary cocrystals. Their shape‐based approach allowed the partial replacement of one component in a binary cocrystal with a structurally similar component to form higher‐order molecular solids.[^42^, ^43^, ^44^, ^45^] This sequential substitution is based on the relative strengths of the HBs present, where only the more robust interactions persist after substitution forming the new cocrystal.

The third strategy—for more complex systems—as demonstrated by Topić and Rissanen^[46]^ involved the combination of synthons comprising robust orthogonal interactions (i.e. interactions that do not interfere with one another)—such as HalB and HBs (***Strategy III***). A series of crown ethers, thioureas and perfluorinated HalB donors were used to produce ternary cocrystals with high orthogonality between the interactions present—demonstrating an effective design‐based strategy to produce complex molecular solids from simple building blocks.

Generally, the synthesis of binary cocrystals is performed in solution. However, there are a number of challenges associated with solution methods for the synthesis of higher‐order phosphazane cocrystals. Firstly, inherent solubility differences between chemical species limit the choice of solvents that enable HOCs. Secondly, to avoid unsolicited solvent molecules within the cocrystal lattice,^[3]^ these choices are further limited.

This challenge, for instance, is demonstrated by the high affinity of phosphazane species for certain solvents as they readily crystallize with a specific solvate molecule despite being in a physical mixture with other components.^[36]^ Furthermore, the use of solution‐based methodologies increases the probability of conglomerate crystallization instead of the desired multicomponent cocrystals.

In contrast to solution methods, mechanochemistry has been successfully employed for *(i)* the rational and targeted synthesis of a range of supramolecular architectures and frameworks,^[47]^ (ii) rapid cocrystal screening and formation,[^48^, ^49^, ^50^] and *(iii)* reproducibly enabling intricate multicomponent cocrystals.[^27^, ^51^, ^52^]

**Scheme 1:**
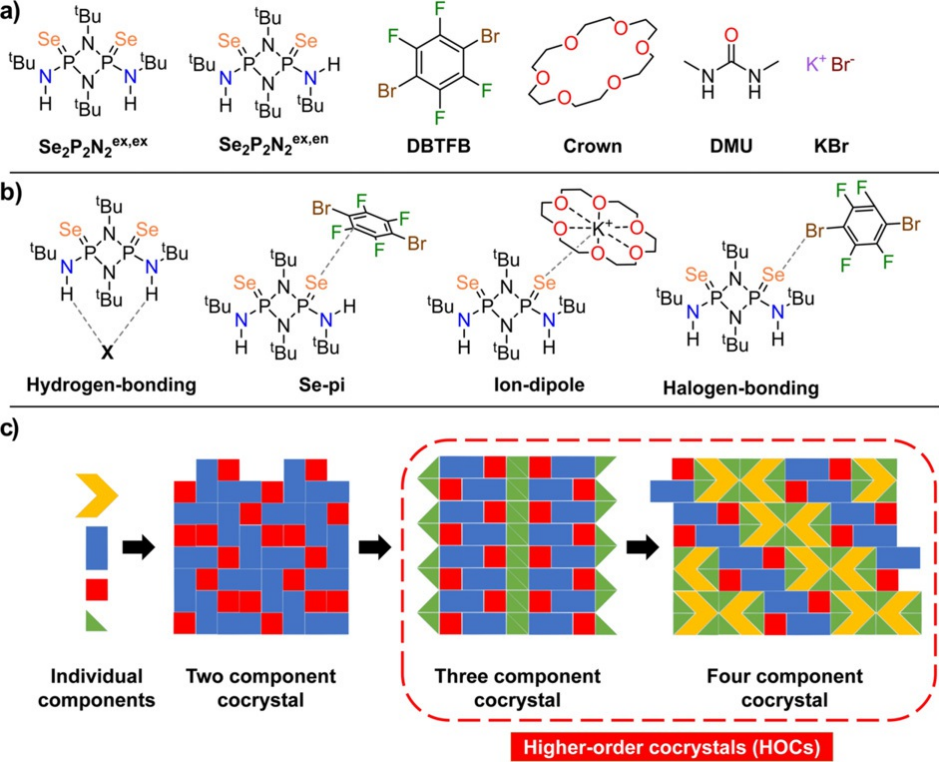
a) Molecular structures involved in the synthesis of cocrystals. b) Supramolecular motifs and interactions displayed by the **Se_2_P_2_N_2_** molecule in the presence of various coformers. c) Schematic illustration of the serial mechanosynthesis approach used for the construction of higher‐order cocrystals (HOCs).

Building on these precedents—*(i)* reported cocrystal synthetic strategies (*Strategies I‐III*), *(ii)* the limited number of HOCs involving complex inorganic building blocks, and *(iii)* the value of mechanochemistry in cocrystal synthesis—we set out to crystal engineer the first novel series of HOCs comprising “complex” cyclophosphazane building blocks by exploiting both intermolecular interaction hierarchy, and synthon orthogonality as design principles for their construction (Scheme 1).

**Scheme 2:**
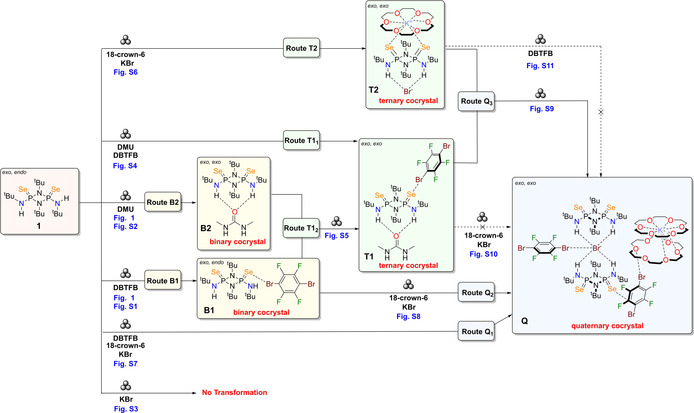
Mechanosynthesis of multicomponent cocrystals based on the **Se_2_P_2_N_2_** (**1**) molecule. The ternary (**T1** and **T2**) and quaternary cocrystal (**Q**) can be obtained sequentially in neat grinding (**Routes T1_2_**, **Q2** and **Q3**) experiments or by direct one‐pot neat grinding (**Routes T1_1_**, **T2** and **Q1**) experiments from the individual components. Detailed breakdown of the formation of the binary, ternary and quaternary cocrystals are shown in Scheme S2, S3 and S4 respectively in the Supporting Information.

Herein, we report serial and one‐pot (i.e., telescopic synthesis)^[53]^ routes for the mechanosynthesis of ternary and quaternary HOCs (see Scheme 2). In contrast to previously reported HOCs, our crystals are based on complex, but easily tunable, inorganic cyclodiphosphazane supramolecular synthons. Moreover, we also demonstrate the formation of ternary and quaternary cocrystals from lower‐order binary and ternary cocrystals as reagents—which is hitherto unreported.

In addition, computational methods were used to estimate the binding modes, stabilization energies and bulk mechanical properties of the resulting HOCs. The results show that careful manipulation of the composition of the HOCs allows us to tune the stability and bulk properties of the materials.

This work creates both new opportunities in hybrid organic‐inorganic molecular solids, and demonstrates the synergy between mechanosynthesis and *in‐silico* computational methods for the rapid screening and selection of HOCs, enabling control over both their functionality and properties.

## Results and Discussion

### Direct Synthesis of Binary Cocrystals (B1 and B2)

Previously, it has been demonstrated that air‐ and moisture‐stable cyclodiphosph(V)azane compounds can be obtained via oxidation of the phosphorus(III) centers to phosphorus(V) using chalcogenic elements.[^35^, ^54^, ^55^] Within the *cis*‐oxidized species, the terminal substituents can adopt several topological arrangements giving rise to two conformations (Scheme 1). Of particular interest is the *exo*, *exo* (Z, Z) conformation, which is a versatile bifurcated hydrogen bond (HB) donor, due to the presence of two converging terminal NH groups. Seminal work from Goldfuss et al. demonstrated that these species are selective towards halide and acetate recognition via R^2^_1_(8) bifurcated HB interactions.^[39]^ This approach has recently been used in the synthesis of a range of versatile monomeric, dimeric and trimeric halide hosts.^[33]^

In solution, the symmetrically substituted *cis*‐Se oxidized cyclodiphosph(V/V)azane comprising four *tert*‐butyl groups—[^*t*^BuNH(P(Se)μ‐N^*t*^Bu)]_2_, (**Se_2_P_2_N_2_**)—has been reported to cocrystallize with N, N′‐dimethylurea (**DMU**) and 1,4‐dibromotetrafluorobenzene (**DBTFB**) in a 1:1 ratio to form binary (**Se_2_P_2_N_2_**)⋅(**DBTFB**) and (**Se_2_P_2_N_2_**)⋅(**DMU**) cocrystals (**B1** and **B2**, respectively).^[36]^

Building block **Se_2_P_2_N_2_** (**1**), which adopts an *exo*, *endo* (*Z*, *E*) conformation (CSD code **XOTGAO**)^[56]^ rearranges to an *exo*, *exo* (*Z*, *Z*) conformation, **Se_2_P_2_N_2_** ^***exo,exo***^,^[57]^ upon the formation of R^2^_1_(8) bifurcated HBs with **DMU** in **B2**. Binary cocrystal **B2** can be isolated in two different polymorphic forms (i.e., **B2‐*Form I*** and **B2‐*Form II***, *Pna*2_1_ and *P*2_1_/*c*, respectively).

In **B1**, **Se_2_P_2_N_2_** adopts an *exo*, *endo* (*Z*, *E*) conformation (**Se_2_P_2_N_2_** ^***exo,endo***^, Scheme 1)—which is identical to the **XOTGAO** structure (vide supra). This is attributed to the absence of strong HB acceptors which is the driving force for adopting the *exo*, *exo* (*Z*, *Z*) conformation. The asymmetric nature of **Se_2_P_2_N_2_** ^***exo,endo***^ results in **B1** HalBs and Se⋅⋅⋅π interactions with **DBTFB** molecules on the *exo* and *endo* sides, respectively (see SI).

The calculated packing indices (PI) are 63.30 %, 65.0 %, and 66.30 % for **B2‐*Form I***, **B2‐*Form II***, and **B1**, respectively (Table 1). Notably, **B2** polymorphs display solvent‐accessible voids in the crystals (0.59 % and 3.74 % of the unit cell volume in ***Form I*** and for ***Form II***, respectively, Table 1).

**Table 1:** Properties of the cocrystals in this work.^[a]^

Solid Form	Se_2_P_2_N_2_ Conformation	Crystal density [g cm^−3^]	PI [%]	*V*_void_ [%]	Δ*E*_comp_ [kJ mol^−1^]	Δ*E*_coc_ [kJ mol^−1^]	B [GPA]	β [TPa^−1^]
**B1**	*exo‐endo*	1.73	66.30	0.00	−16.00	−10.00	22.78	46.20
**B2‐** *Form II* (Z′′=4)	*exo‐exo*	1.38	65.00	0.59	−44.04	−3.79	13.88	72.53
**B2‐** *Form I* (Z′′=6)	*exo‐exo*	1.34	63.30	3.74	−41.47	−3.80	9.31	120.46
**T1**	*exo‐exo*	1.51	65.10	3.30	−29.62	−5.07	9.01	141.58
**T2**	*exo‐exo*	1.73	71.40	0.96	−122.48	–	–	–
**Q**	*exo‐exo*	1.62	68.40	0.00	−73.02	–	–	–

[a] Packing index PI (%), *V*_void_=Void cell volume (%), B=Bulk modulus, *β*=Compressibility, Δ*E*_comp_=DFT complexation energy per fragment in the asymmetric unit, Δ*E*_coc_=DFT bulk cocrystal stabilization energy per formula unit. Note: For **T2** and **Q**, the Δ*E*_coc_, the bulk modulus (B) and compressibility (β) could not be computed due to the lack of either reference structures (Δ*E*_coc_) or suitable potentials (B and β) for computing these properties.

**Figure 1:**
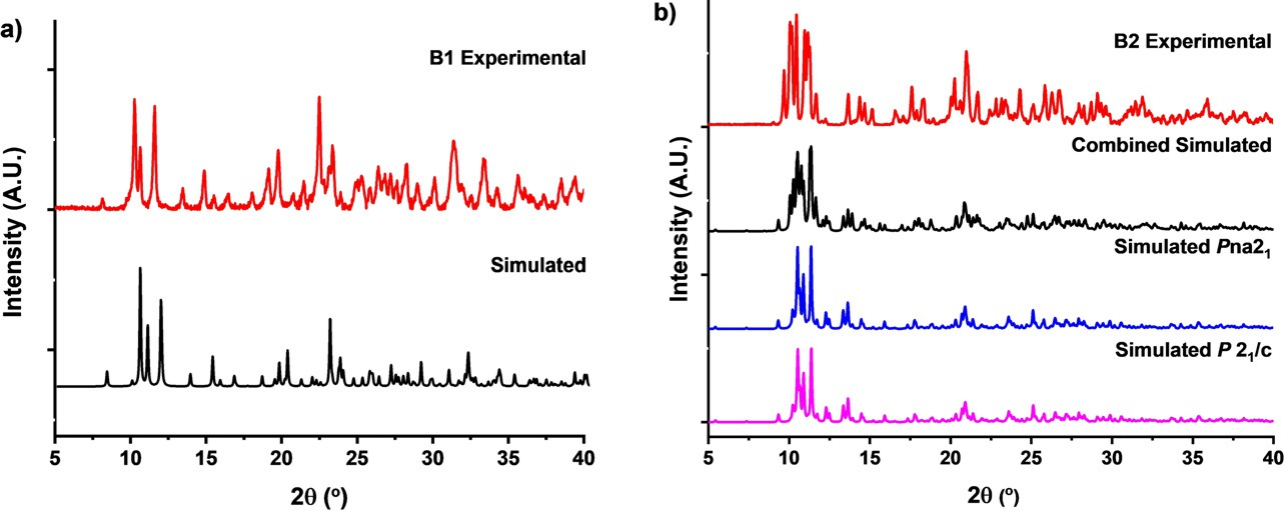
Overlay of powder diffraction patterns of the mechanochemically obtained powders and simulated patterns from single‐crystal data of the binary cocrystals of **Se_2_P_2_N_2_** and a) **DBTFB**, b) **DMU**; two polymorphs of the binary cocrystal were obtained. For expanded view see Supporting Information.

Our studies began by investigating the formation of these species via grinding the individual components, which produced microcrystalline powders of both cocrystals within 30 min, as evidenced by powder X‐ray diffraction (PXRD) patterns (Figure 1). Comparison of the diffractograms of the resultant solids with that of the individual components showed no trace of the starting materials (Figures S1,S2) and the pattern matched those simulated from single‐crystal x‐ray diffraction (SCXRD) data.

This provided the motivation for the construction of more complex assemblies. Hence, rational hierarchical intermolecular interactions and orthogonal synthons were judiciously combined to furnish higher‐order ternary and quaternary cocrystals (vide infra).

### Serial and One‐pot Mechanosynthesis of Ternary cocrystal 1 (T1): (Se_2_P_2_N_2_)⋅(DMU)⋅(DBTFB)

Since **Se_2_P_2_N_2_** can engage in HalB and HB interactions at different sites (i.e., with Se and NH moieties, respectively) as shown in **B1** and **B2**, respectively, we hypothesized that ternary cocrystals could be engineered using **Se_2_P_2_N_2_**, **DMU** and **DBTFB** using synthon orthogonality to generate hybrid HOCs.

**Figure 2:**
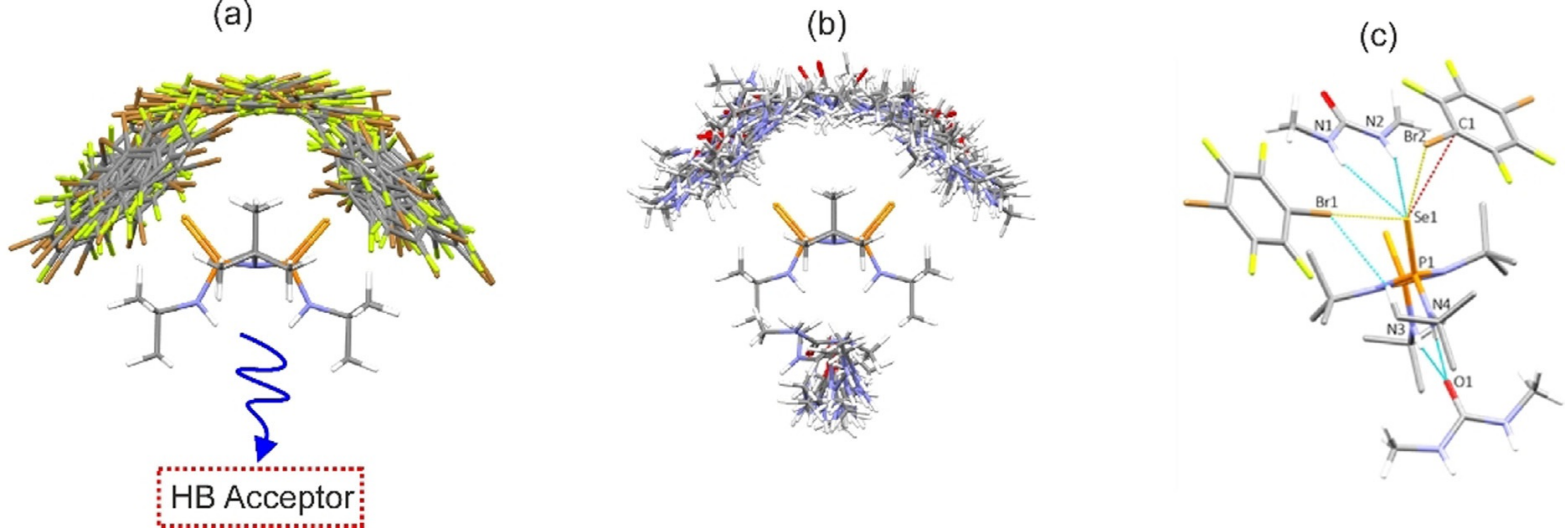
Monte Carlo simulated trajectories for the 100 most‐stable binding geometries between **Se_2_P_2_N_2_** ^***exo,exo***^ and either a) **DBTFB** or b) **DMU**. c)  Portion of the experimentally determined packing in ternary cocrystal **T1** depicting the Se⋅⋅⋅Br, Se⋅⋅⋅H−N and Se⋅⋅⋅π interactions. Some C−H hydrogens are omitted for clarity.

Such a site‐selective bifunctionality was confirmed by initial Monte Carlo simulations, which suggested that **Se_2_P_2_N_2_** can form binary complexes with **DBTFB** via Se⋅⋅⋅π or Se⋅⋅⋅Br interactions (Figure 2 a), leaving the NH HB donors on **Se_2_P_2_N_2_** free to engage in further intermolecular HB interactions. Simulations using **DMU** as the coformer also suggest that stable complexes with **Se_2_P_2_N_2_** can be formed via the formation of Se⋅⋅⋅H‐N or NH⋅⋅⋅O interactions between the two molecules (Figure 2 b). Hence, we postulated that if both **DBTFB** and **DMU** were used in cocrystallization experiments with **Se_2_P_2_N_2_**, these synthons would not compete with one another (orthogonality).

Indeed, milling **Se_2_P_2_N_2_**, **DMU**, and **DBTFB** in a 2:2:1 ratio at 30 Hz for 30 min—direct one‐pot **Route T1_1_** in Scheme 2—afforded a new molecular solid powder—with almost quantitative conversion of the individual components to the new crystalline product. This is evident from the PXRD patterns obtained (Figure S4, and SI Section 1.3). Diffraction quality single crystals of the (**Se_2_P_2_N_2_**)**⋅**(**DMU**)**⋅**(**DBTFB**), (**T1**) neutral ternary cocrystal, were obtained by slow evaporation of the reaction mixture in methanol. The simulated PXRD pattern obtained from the single crystal data matched those of the powders obtained mechanochemically. Analysis of the crystal structure of **T1** revealed that the bifurcated hydrogen‐bonding of **Se_2_P_2_N_2_** with **DMU** persists, along with Type II bifurcated halogen‐bonding interactions via the ditopic **DBTFB** molecule (Figure 2 c).^[58]^

Our Monte Carlo generated interactions show a strong agreement with the observed intermolecular interactions in **T1**. The preferential “top face” binding of **DBTFB** with **Se_2_P_2_N_2_** in all the Monte Carlo binding trajectories is consistent with the crystal packing in **T1**, which shows that only Se⋅⋅⋅π or Se⋅⋅⋅Br interactions are observed between these molecules. Consequently, the oxygen of **DMU** is able to engage in a bifurcated HB with the unused NH donors of **Se_2_P_2_N_2_**.

The orthogonal interplay between HBs and HalBs resembles cocrystals involving thioureas.^[46]^ However, in **T1**, one of the Se atoms of the **Se_2_P_2_N_2_** can simultaneously undergo three different types of intermolecular interactions: (i) HalB with **DBTFB** (Se⋅⋅⋅Br distance ca. 3.42 Å), (ii) bifurcated HBs with **DMU** (Se⋅⋅⋅H‐N distance ca. 3.71 Å), and (iii) long range Se⋅⋅⋅π interaction to the electron deficient **DBTFB** molecule (Se⋅⋅⋅C distance ca. 3.43 Å)—also observed in the **B1** binary cocrystal.

Notably, the ability of Se atoms to simultaneously accommodate several supramolecular interactions—to the best of our knowledge—has not been previously reported. This is attributed to both the large size of the Se atom (ca. 1.64 Å^3^) and the electron rich cyclodiphosphazane P_2_N_2_ backbone, where electron density can delocalize onto the P=Se bond, enabling simultaneous interactions. This underscores the unique nature of Se‐oxidized phosphazanes as versatile inorganic building blocks to construct complex supramolecular assemblies.

Whilst the formation of binary cocrystals via mechanochemistry has previously been described, the factors dictating their transformation into higher‐order ternary and quaternary crystals is poorly understood. Hence, the obtained ternary cocrystal (**T1**) presents a unique proof‐of‐concept opportunity to assess whether ball‐milling enables the synthesis of HOCs using a lower‐order system (i.e., binary crystals) as supramolecular reagents—which to the best of our knowledge has never been demonstrated for hybrid cocrystals comprising the **Se_2_P_2_N_2_** core.

Indeed, milling binary cocrystals of **B1** and **B2** in equimolar amounts—sequential **Route T1_2_** in Scheme 2 (see Figure S5) produces the desired **T1** cocrystal. The use of binary cocrystals as reagents to form HOCs cannot easily be achieved in solution, but can be readily realized in the solid‐state, which demonstrates mechanochemistry as a powerful synthetic tool to obtain complex multicomponent solids.

The synthesis of **T1** from either binary cocrystal precursors or individual components demonstrates the synthetic flexibility and potential of **Se_2_P_2_N_2_** to form robust and reproducible supramolecular synthons.

**Figure 3:**
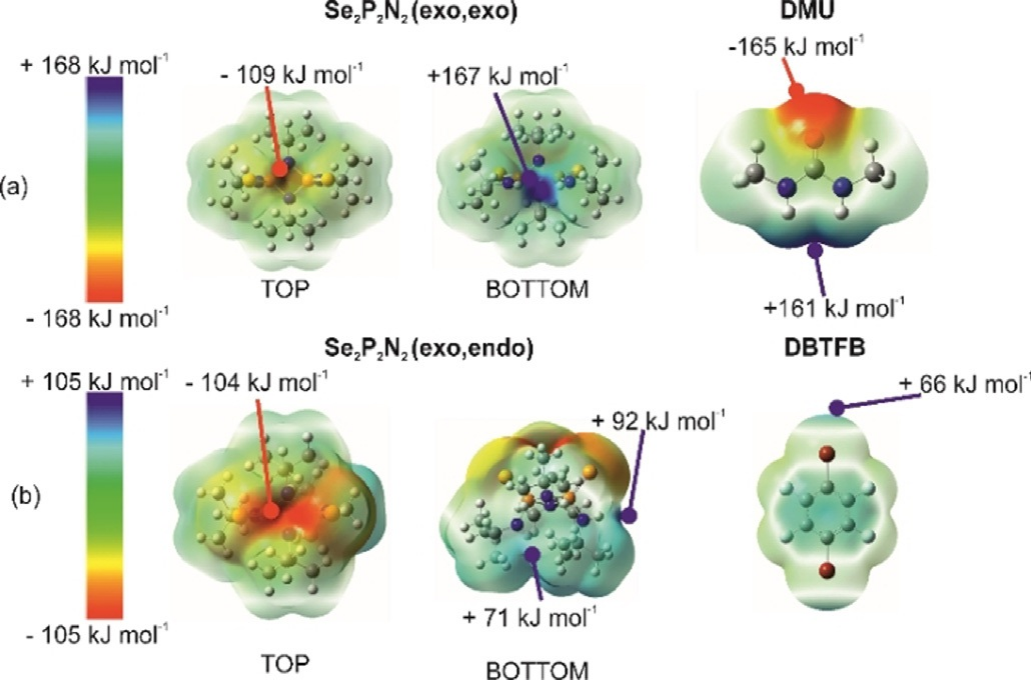
Molecular electrostatic potential (MEP) surfaces for a) **Se_2_P_2_N_2_** (top and bottom views) and **DMU**; b) **Se_2_P_2_N_2_** (top and bottom views) and **DBTFB**. See Supporting Information for expanded version.

The calculated molecular electrostatic potentials (MEPs, Figure 3) support the observed heterosynthons in the cocrystals. **DMU** engages in directional HB interactions with **Se_2_P_2_N_2_** in **B2** and **T1** because of the large negative potential (i.e., −165 kJ mol^−1^) on the carbonyl acceptor group. In contrast, the bromines on **DBTFB** are associated with a positive potential of +66 kJ mol^−1^, thereby facilitating the formation of complementary Se⋅⋅⋅Br interactions in **B1** and **T1**. The observed electron density distribution in **Se_2_P_2_N_2_** is strongly dependent on the molecular conformation. The *exo*,*exo* conformation has a strong positive potential of approximately +167 kJ mol^−1^ due to converging NH donors, whereas the *exo*,*endo* conformation is a weaker HB donor as indicated by the comparable potentials of +71 kJ mol^−1^ and +92 kJ mol^−1^ at each of the divergent NH donor moieties (*endo* and *exo* positions, respectively—Figure 3).

In line with previous reports,^[33]^ this variation in the HB donor capacity of **Se_2_P_2_N_2_** explains the observed **Se_2_P_2_N_2_** ^***exo,exo***^ conformation in all cocrystals containing **DMU** (**B2** *‐Form I*, **B2**‐*Form II* and **T1**) since it enables the formation of bifurcated HB interactions with the oxygen acceptor of **DMU**. The calculated MEPs also support the observed orthogonality of hydrogen‐ and halogen‐bonding interactions in ternary cocrystal **T1**. This is evident from the MEPs as the NH donors on **Se_2_P_2_N_2_** are more likely to be engaged in bifurcated HB interactions with the carbonyl acceptor of **DMU** (i.e. the strongest pairing according to the calculated potentials), allowing the Se atoms to freely engage in Se⋅⋅⋅Br interactions with **DBTFB**.

### Direct Synthesis of Ternary cocrystal 2 (T2): (Se_2_P_2_N_2_)⋅(KBr)⋅(18‐crown‐6 ether)

**Figure 4:**
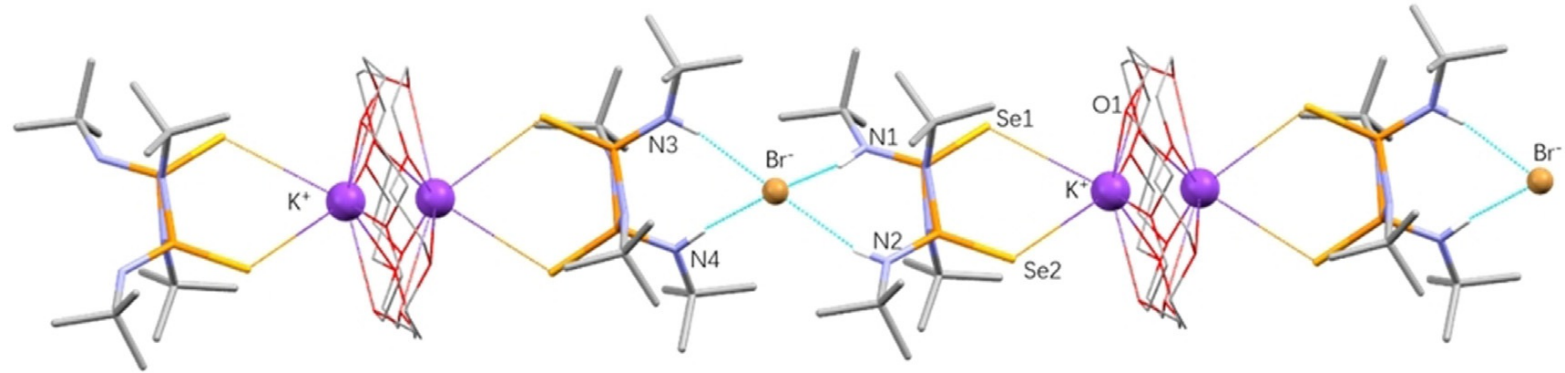
Portion of the experimentally determined packing in the crystal structure of the ternary ICC, (**Se_2_P_2_N_2_**)⋅(**crown**)⋅(**KBr**) **T2**, forming one‐dimensional chains linked by Se⋅⋅⋅K^+^ and Br^−^⋅⋅⋅H−N interactions. The crown and K^+^ are positionally disordered over two sites. Some C−H hydrogens are omitted for clarity. For expanded version see Supporting Information.

Given the proven ability of cyclodiphospha(V/V)zane species to coordinate metal cations (via the formation of Se‐M coordinative bonds)^[58]^ as well as to bind to halides (via bifurcated HB N−H⋅⋅⋅X^−^ interactions),^[54]^ we envisaged the formation of ternary ICCs[^14^, ^15^] by combining **Se_2_P_2_N_2_** with simple atomic salts (such as KBr)—see Figure 4.

Initial screening experiments by grinding **Se_2_P_2_N_2_** with **KBr**, however, did not result in any changes of the powder pattern, suggesting the formation of a physical mixture (Figure S3). Hence, 18‐crown‐6 ether (**crown**) was added, a commonly used strategy in organic chemistry for trapping K^+^ (and other alkali) metal cations.[^18^, ^59^] Under optimized conditions, milling **Se_2_P_2_N_2_**, **crown** and **KBr**, in a 2:1:1 molar ratio for three hours—direct one‐pot **Route T2** in Scheme 2—successfully led to the desired ICC, (**Se_2_P_2_N_2_**)**⋅**(**crown**)**⋅**(**KBr**) (**T2**). Diffraction quality crystals of **T2** were subsequently obtained by slow evaporation from solution using either CHCl_3_ or isopropanol as the solvent.

Cocrystal **T2** comprises a one‐dimensional catemer where the K^+^ cation is coordinated to a crown ether moiety and a **Se_2_P_2_N_2_** unit acting as a chelating ligand through the P=Se bonds (Se⋅⋅⋅K^+^ 3.75 Å).[^58^, ^60^] The complexed K^+^ crown portion is disordered over two sites along the chains. On the other hand, the Br^−^ counter anion is stabilized by two sets of bifurcated charge‐assisted hydrogen‐bonds (CAHyB) via four N‐H moieties from two **Se_2_P_2_N_2_** molecules (N−H**⋅⋅⋅**Br^−^ 3.40 Å)^[61]^ within this chain.

### One‐pot and sequential Mechanosynthesis of Quaternary cocrystal (Q)

The formation of quaternary and other HOCs can be very challenging especially in systems involving a large variety of functionalities that can form different combinations of intermolecular interactions, rendering the prediction of any supramolecular synthon hierarchy taxing.[^62^, ^63^] Therefore, we set off to explore the synthesis of higher‐order quaternary cocrystals using our mechanochemical approach, supported by computational modelling (vide infra).

Ball‐milling **Se_2_P_2_N_2_**, **DBTFB**, **crown** and **KBr** in 2:2:2:1 and 2:2:1:1 molar ratios for 30 min at 30 Hz—direct one‐pot **Route Q_1_** in Scheme 2—resulted in the formation of a microcrystalline powder, whose PXRD pattern contained new peaks at low angles which indicate a new solid form containing a large unit cell had formed (Figure S7). Diffraction quality crystals were obtained by slow evaporation of the reaction mixture in THF.

**Figure 5:**
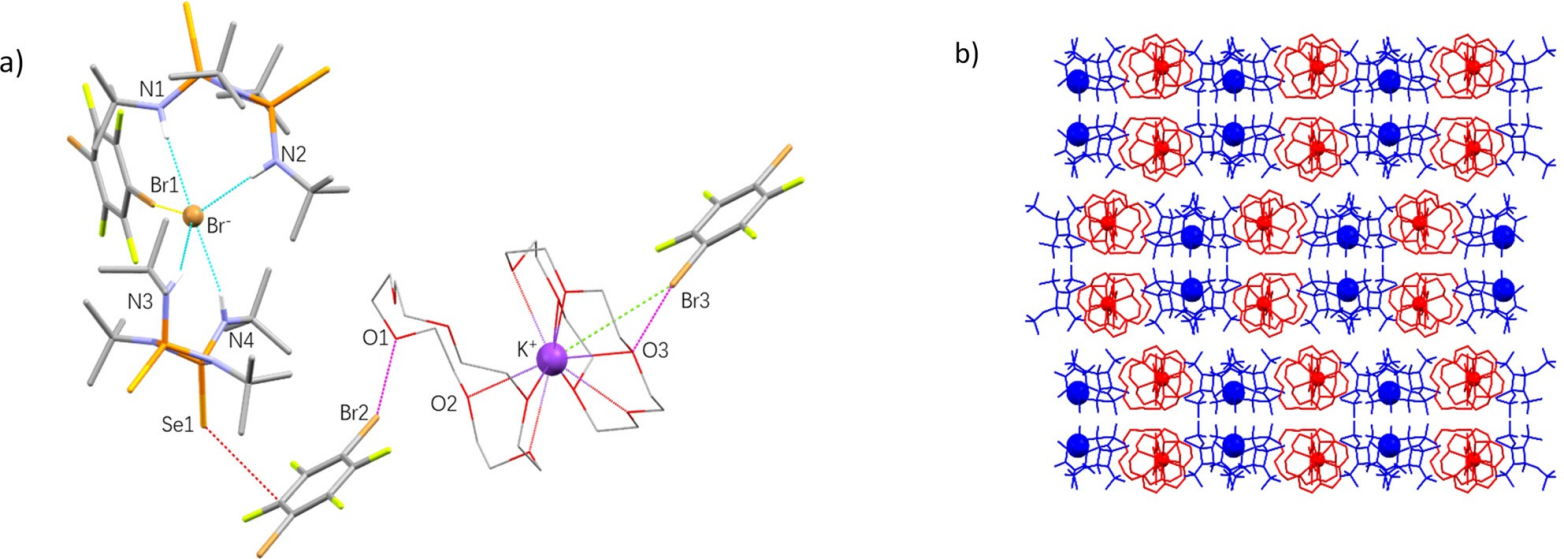
a)Portion of the experimentally determined crystal structure of the four‐component ICC, (**Se_2_P_2_N_2_**)_2_⋅(**DBTFB**)_2_⋅(**crown**)_2_⋅(**KBr**) (**Q**), depicting the N−H⋅⋅⋅Br^−^, O⋅⋅⋅Br, Br⋅⋅⋅Br^−^, O⋅⋅⋅K^+^ and Se⋅⋅⋅π interactions; b)  Portion of the experimentally determined crystal structure of the four‐component ICC, **Q**, depicting the packing motif as viewed down the crystallographic *c*‐axis. The red and blue domains are linked by Se⋅⋅⋅π interactions. The blue spheres are the Br^−^ anions and the red spheres are the K^+^ cations. For expanded version see Supporting Information.

SCXRD analysis confirmed the formation of a quaternary ICC (**Se_2_P_2_N_2_**)_2_⋅(**DBTFB**)_2_⋅(**crown**)_2_⋅(**KBr**), (**Q** in Figure 5). The quaternary ICC revealed several intermolecular interactions, which provide insights into the supramolecular hierarchy within our system. Firstly, the robust bifurcated HB synthon of the **Se_2_P_2_N_2_** was retained, and similar to the **T2** cocrystal, the Br^−^ is stabilized by four CAHyB N−H⋅⋅⋅Br^−^ interactions from two **Se_2_P_2_N_2_** molecules. Closer inspection of the intermolecular interactions revealed that the Br^−^ also undergoes Type I halogen‐bonding^[18]^ with a molecule of **DBTFB**. Such a Br⋅⋅⋅Br^−^ interaction is reminiscent of charge‐assisted halogen‐bonds (CAHalB) that was previously observed by several groups.^[64]^

Another expected interaction is the formation of ion‐dipole interactions between K^+^ and two **crown** molecules, albeit one of the **crown** molecules not fully encapsulating the cation. Consequently, the formation of this “half‐crown” complex creates coordinatively available ether sites, allowing for O⋅⋅⋅Br HalBs (2.94–2.99 Å) to be formed with neighboring **DBTFB** molecules. The ditopic **DBTFB** molecules and the bis‐**crown‐K** ^+^ complex formed linear catemers linked by bidirectional HalBs. The presence of strong O⋅⋅⋅Br halogen bonds could also explain the absence of the weaker Se⋅⋅⋅Br HalB (ca. 3.42 Å) in this cocrystal, illustrating the preferential hierarchy of O⋅⋅⋅Br vs. Se⋅⋅⋅Br supramolecular interactions.^[20]^ However, despite the lack of Se⋅⋅⋅Br HalBs, the two crystallographically inequivalent **DBTFB** molecules still form Se⋅⋅⋅π interactions with the **Se_2_P_2_N_2_** molecules present. These Se⋅⋅⋅π interactions (ca. 3.48–3.57 Å) have also been observed in all cocrystals involving **Se_2_P_2_N_2_** and **DBTFB** (two‐, three‐ and four‐component crystals), indicating their robustness and high reproducibility as supramolecular synthons.

The packing in **Q** can be divided into two distinct domains (Figure 5 b). The first (in red) consists of a Br^−^ anion with two molecules of **Se_2_P_2_N_2_** and one **DBTFB** molecule, stabilized by CAHyB and CAHaB interactions, respectively. The second (in blue) is composed of the K^+^ cation encapsulated by two **crown** molecules, which form halogen‐bonded chains with another molecule of **DBTFB**. Both domains form alternating layers and are linked together by the two Se⋅⋅⋅π intermolecular interactions as they propagate along the crystallographic *c*‐axis.

To further test our stepwise approach for the mechanosynthesis of HOCs, several alternative routes were explored for the preparation of **Q**. Firstly, milling a mixture of the previously obtained binary cocrystal **B1**, **crown** and **KBr**—sequential **Route Q_2_**—successfully produced the target quaternary cocrystal **Q** as indicated by the PXRD pattern of the obtained crystalline solid (Figure S8). Regrettably, despite several attempted reactions using cocrystals **T1** and **T2** as starting materials, the desired quaternary cocrystal **Q** could not be obtained (see Scheme 2 and Figures S10,S11).

Finally, the stepwise approach was also tested for the synthesis of **Q** by milling together both ternary cocrystals **T1** and **T2**. Notably, the milling of both ternary cocrystals **T1** and **T2** in a 1:1 ratio—sequential **Route Q_3_** in Scheme 2—yielded a mixture of the desired cocrystal **Q** and **DMU** as indicated by the PXRD pattern of the obtained crystalline powder (Figure S9).

Overall, our experimental results show that binary and HOCs based on **Se_2_P_2_N_2_** can be readily obtained from their single individual components, or lower‐order (i.e., binary and ternary) crystal building blocks via both direct and/or one‐pot mechanochemical transformations.

### Energetic considerations and predicted mechanical properties

The systematic crystal engineering of HOCs requires a fundamental understanding of the driving forces behind their formation. Generally, binary cocrystals are thermodynamically driven as shown in an extensive survey of 350 binary cocrystals comprising both HB and HalB interactions.^[65]^ On average, more than 95 % of the surveyed cocrystals were more stable than the stoichiometrically weighted sum of their component crystals with an average stabilization energy of −8 kJ mol^−1^. In contrast, for HOCs such as **T1**, **T2** and **Q** much less is known about the energetics behind their formation.

Recent work on molecular ICCs comprising HBs has shown that their structures can be reliably predicted using computational methods due to the presence of recurring CAHyB interactions.^[66]^ However, the mean stabilization energy for these species was found to only be −2 kJ mol^−1^, which is significantly less stable than that of binary cocrystals.^[67]^ This suggests that factors other than thermodynamics may be important in determining which set of binary cocrystals can be used to construct ternary and quaternary cocrystals.

To understand the formation of the reported cocrystals (Table 1), two types of DFT calculations were performed: (i) complexation energies (Δ*E*_comp_) between the unique chemical species in the crystals ignoring packing effects and where possible, (ii) bulk cocrystal (Δ*E*_coc_) stabilization energies considering the crystal packing forces.

**Figure 6:**
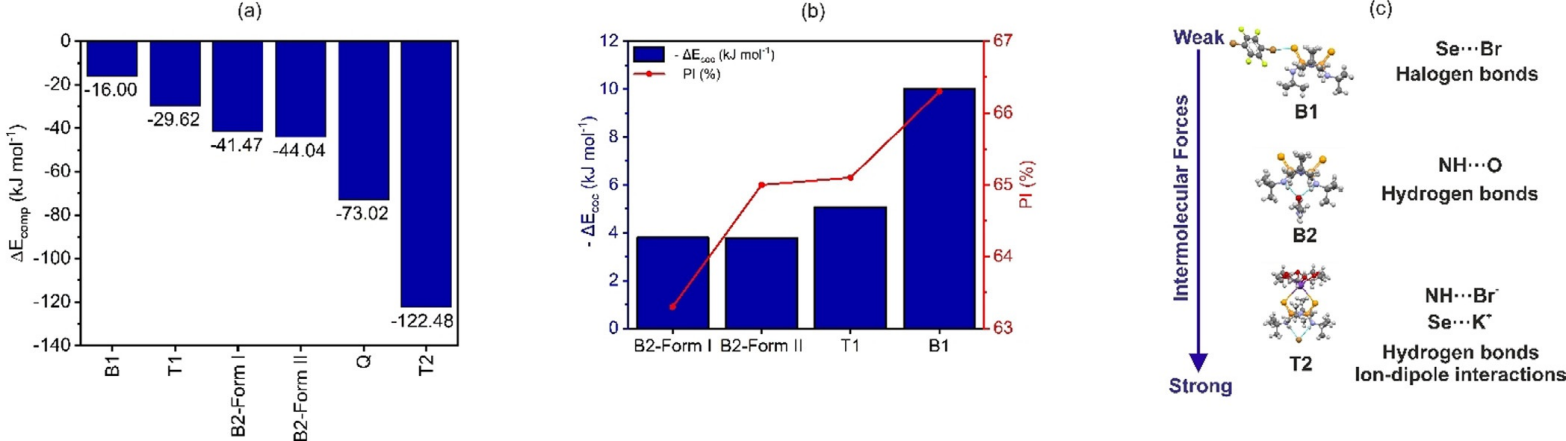
a) Bar graph of the trend in the complexation energy (Δ*E*_comp_) for each cocrystal. b) Variation in the DFT energy for the formation of the molecular cocrystals (−Δ*E*_coc_) **B1**, **B2** and **T1** with the packing index (PI) of each cocrystal. c) Illustration of the observed synthons and relative strengths of the intermolecular interactions observed in the various cocrystals. For expanded version see Supporting Information.

The computed Δ*E*_comp_ energies show a clear thermodynamic driving force for molecular association between the components in each cocrystal, ranging from −16 kJ mol^−1^ for **B1** to −122 kJ mol^−1^ for **T2** (see Figure 6 a and Table 1).

Moreover, a general trend of increasing stabilization going from binary to quaternary cocrystals is observed, which agrees with the hierarchy of supramolecular synthons. **B1** displays the least stabilizing complexation energy due to the weaker Se⋅⋅⋅Br heterosynthons as compared to the NH⋅⋅⋅O=C HBs observed in both **B2** and **T1** (Figure 6 a and c). In contrast, **T2** displays the most stabilizing complexation energy of all species due to the favorable multi‐center coulombic interactions in the ICC. This is evidenced by the K^+^ metal ions coordinating to both **Se_2_P_2_N_2_** and the crown ether, leading to more stabilizing complexation energies in **T2** when compared to **Q** where the **Se_2_P_2_N_2_** molecules are not coordinated around the K^+^ ions.

When crystal packing effects are considered, DFT estimates of Δ*E*_coc_ (Table 1) suggest that the efficiently packed **B1** cocrystal (PI: 66.30 %) displays the highest stabilization energy (Figure 6 b and Table 1) of −10 kJ mol^−1^ relative to the stoichiometrically weighted sum of the component crystal energies. In contrast, the two polymorphs of **B2** (i.e., **B2*‐Form I*** and ‐***Form II***, Z′′=6 and 4 respectively) are equi‐energetic (−3.80 kJ mol^−1^) when crystal packing forces are considered, with no energetic preference between the crystal forms (Figure 6 b). This is due to the comparable packing forces of the molecules in the two forms as they display the same interactions between **DMU** and **Se_2_P_2_N_2_** but differ only in the number of symmetrically inequivalent units of **DMU** and **Se_2_P_2_N_2_** within the crystal. When compared to **B1**, the two polymorphs of **B2** display the lowest PI of all the cocrystals obtained. The molecular ICC **T1** is also predicted to be thermodynamically favored to form (Figure 6 b and Table 1), according to the calculated Δ*E*_coc_ of −5.07 kJ mol^−1^. For **T2** and **Q**, the absence of reference component crystal structures prevented computing Δ*E*_coc_.

One of the motivations for the discovery of HOCs is to widen the range of solid‐state properties adopted by a reference molecule when formulated in different solid forms. This is enabled by changes to the chemical composition of the crystals, which affect the resulting bulk material properties.^[[68]]^ Mechanical properties are an example of a solid‐state property that are critical to the performance characteristics of solid materials.

To understand how the stoichiometry and intermolecular forces affect the mechanical properties of the reported cocrystals, we computed their bulk modulus (B) and compressibilities (β). Their predicted mechanical properties vary significantly, depending on the types of interactions present (Table 1). The halogen‐bonded **B1** displays a bulk modulus of 22.78 GPa, which contrasts with the 13.88 GPa predicted for the hydrogen‐bonded **B2‐*Form II***. The less efficiently packed **B2‐*Form I*** displays a bulk modulus of 9.31 GPa whilst **T1** displays a bulk modulus of 9.01 GPa.

The computed compressibilities are correlated with the packing efficiencies of the components in the crystals. **B2‐Form I** and **T1** both display large β values that are >100 TPa^−1^, reflecting the poor packing in the cocrystals with void space accounting for more than 3 % of the unit cell volumes. In contrast, the efficiently packed, highly dense **B2‐*Form II*** and **B1** crystals display β values that are <80 TPa^−1^.

The computed DFT complexation and bulk stabilization energies suggest favorable thermodynamics for forming HOCs. The variations in the computed mechanical properties of these species reflect the differences in the intermolecular forces binding the chemical fragments together and suggest that careful choice of the coformers can be used to tune the properties of the cyclodiphosphazane cocrystal.

## Conclusion

In summary, this study has shown how mechanochemistry can be used to access binary and higher‐order multicomponent cocrystals via both serial and one‐pot mechanosynthesis routes (i.e., from individual components, as well as from lower‐order counterparts)—which cannot readily be conducted using conventional solution methods.

Mechanochemistry, in this case, presents a rapid, convenient, and efficient route to afford the targeted HOCs, whilst circumventing the problems typically associated with solution‐based chemistry such as the differential solubility of the reagents.

Our proof‐of‐concept mechanochemical reactions demonstrate how binary cocrystals can be used as reagents to form HOCs in the solid‐state, and further underscores mechanochemistry as a powerful synthetic tool to obtain complex multicomponent solids.

Notably, the formation of ternary and quaternary cocrystals is enabled by the ability of the phosphazane building block to simultaneously form multiple robust supramolecular synthons (i.e., bifurcated HBs, HalBs, Se**⋅⋅⋅**π and ion‐dipole interactions). Furthermore, each building block in the complex quaternary cocrystal is structurally unique, having great potential for further expansion if each component's isostructurality is considered.

Computational modelling indicates that all HOCs are thermodynamically driven to form, and their mechanical properties vary according to the chemical composition as well as the strength of the intermolecular forces present in the cocrystals. These observations suggest the potential to optimize bulk material properties via the synthesis of HOCs via the precise selection of the chemical constituents within the crystals.

Our work underscores the topological flexibility of phosphazane main group frameworks in enabling access to complex molecular solids. We hope that these results act as a platform for future development of more elaborate phosphazane building blocks towards the synthesis of more complex higher‐order supramolecular arrangements and phosphazane‐based hybrid organic‐inorganic functional materials.

## Supporting information

As a service to our authors and readers, this journal provides supporting information supplied by the authors. Such materials are peer reviewed and may be re‐organized for online delivery, but are not copy‐edited or typeset. Technical support issues arising from supporting information (other than missing files) should be addressed to the authors.

Supporting Information
