# Supplementary material for: Mechanosynthesis of Higher‐Order Cocrystals: Tuning Order, Functionality and Size in Cocrystal Design
Source: Angew Chem Int Ed Engl. 2021 Jul 1;60(32):17481–90. doi: 10.1002/anie.202101248 (PMC8362154; doi:10.1002/anie.202101248)
Supplement: Supplementary file 1 — Supporting Information [file ANIE-60-17481-s001.pdf]

Angewandte Chemie
Eine Zeitschrift der Gesellschaft Deutscher Chemiker

Supporting Information

Mechanosynthesis of Higher-Order Cocrystals: Tuning Order, Functionality and Size in Cocrystal Design**

Zi Xuan Ng+, Davin Tan+, Wei Liang Teo, Felix León, Xiaoyan Shi, Ying Sim, Yongxin Li, Rakesh Ganguly, Yanli Zhao, Sharmarke Mohamed, and Felipe García**

anie_202101248_sm_miscellaneous_information.pdf

Table of Contents

1. Experimental and Computational Methods 1

2. Powder X-Ray Diffraction Patterns **7**

2.1 Binary Cocrystals 8
2.2 Ternary Cocrystals 12
2.3 Quaternary Cocrystals 16

3. Spectroscopic Data for Crystals **21**

4. Single Crystal X-ray Data of Crystals 1-4 **27**

5. Expanded Manuscript Figures **31**

6. References **34**

1. Experimental Methods

$^{31}P\{^1H\}$ ^1H and ^{13}C NMR spectra were recorded on a BRUKER AVANCE III 300 MHz with chemical shifts (δ) given in parts per million (ppm). Deuterated $CDCl_3$ was used as the NMR solvent.

1.1 Synthesis of [^{t}NH(P(Se)μ-NtBu)]$_2$ ($Se_2P_2N_2$) (1):

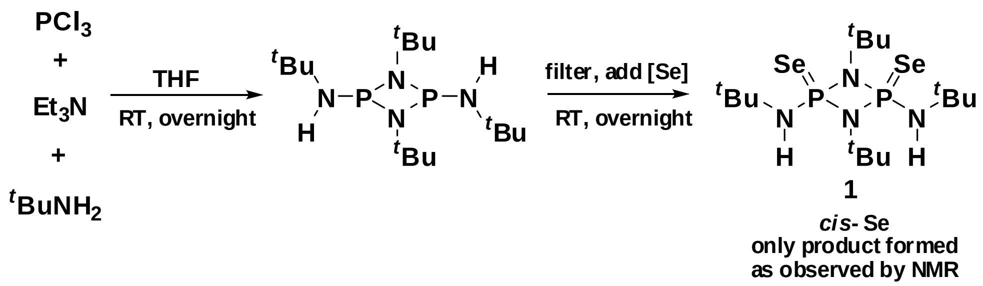

Scheme 2. Synthesis of of [^{t}NH(P(Se)μ-NtBu)]$_2$ ($Se_2P_2N_2$), (1)

5 mmol of distilled PCl_3 and 10mmol of distilled Et_3N was added to a 500 mL two neck round bottom flask containing 250 mL of dry THF as the solvent. The flask was charged with Ar gas and cooled using a mixture of dry ice in acetone. Another solution mixture containing 10mmol of distilled $^tBuNH_2$ in 50 mL of dry THF was added drop wise to the previous mixture *via* a dropping funnel, over a period of 1 hour. Formation of a white precipitate was observed. The reaction was then allowed to stir overnight at room temperature. Next, the reaction mixture was filtered to remove the white solids and the filtrate was transferred to a 350 mL Schlenk flask and the solvent was removed *in vacuo* until the mixture was about 150 mL. 11 mmol of elemental Se was added to the solution and allowed to stir overnight at room temperature. Excess unreacted Se was removed by filtration and the filtrate was collected and transferred to a 250 mL round bottom flask. The remaining solvent was removed using a rotator evaporator and the desired pure product, compound **1** was obtained as an off-white solid.

1.2 Single crystal X-ray diffraction studies

Diffraction-quality crystals were obtained by slow evaporation of solvent from solution in various solvents at room temperature. Data were measured at 103 K with a Bruker Kappa diffractometer equipped with a CCD detector, employing Mo K α radiation (λ = 0.71073 Å), with the SMART suite of programs. Structural solution and refinement were carried out with SHELCTL suite of programs. The structure was solved by direct methods or Patterson maps to locate the heavy atoms, followed by difference maps for the light, non-hydrogen atoms. All non-hydrogen atoms were refined with anisotropic thermal parameters. Crystal **3** and **4** crystallises with two chemically identical but crystallographically unique molecular positions, one with the presence of disordered molecules of 18-crown-6 ether and 1,4-dibromotetrafluorobenzene.

1.3 Powder X-ray diffraction studies

Powder X-ray diffraction (PXRD) data were collected at 40 keV and 15 mA on a Rigaku MiniFlex 600 Benchtop diffractometer using Cu-K*α* radiation (λ = 1.5418 Å) over 2 θ range of 5.0º-50.0º at room temperature. Baseline correction was done using Jade5.

As is common in the field, the outcome of mechanochemical experiments were determined by performing qualitative comparisons of the PXRD patterns of the ground products with those of the starting reagents or intermediate lower-order cocrystal sets. We also compared the experimental powder patterns following ball milling with the simulated PXRD patterns from the single crystal structures of the cocrystals. In all cases, we have observed that the major Bragg reflections in the ground powder samples match those predicted from the single crystal structures. However, in the absence of Rietveld refinement, we cannot be certain that minor impurities of the starting reactants or transformation products are absent.

1.4 Attenuated Total Reflectance Infrared Spectroscopy

Fourier-Transformed Infrared Radiation (FT-IR) Spectroscopic data were obtained using PerkinElmer Spectrum 100 FT-IR Spectrometer, 8 scans from 4000 cm^{-1} to 650 cm^{-1} in the transmission mode, with a resolution of 16 cm^{-1}.

1.5 Mechanochemical synthesis of cocrystals

All milling experiments were conducted in a 10 mL volume stainless steel milling jar that was carefully dried before use by heating at 150 ^{o}C for 2 h and cooling in a vacuum desiccator. The milling jar was then charged with a 10 mm diameter stainless steel ball bearing, **$Se_2P_2N_2$ (1)** and the corresponding components added and the jar and sealed milled for 30 min. Loading and sealing of the milling jar was performed in a glovebox, under an inert, dry atmosphere.

1.5.1 Synthesis of ($Se_2P_2N_2$)·(DBTFB) (*B1*)

The milling jar was charged with a 10 mm diameter stainless steel ball bearing, **$Se_2P_2N_2$ (1)** and **DBTFB** in a 1:1 ratio added and the jar and sealed milled for 30 min. An off-white crystalline powder was obtained.

1.5.2 Synthesis of ($Se_2P_2N_2$)$_2$·(DMU) (*B2*)

The milling jar was charged with a 10 mm diameter stainless steel ball bearing, **$Se_2P_2N_2$ (1)** and **DMU** in a 1:1 ratio added and the jar and sealed milled for 30 min. An off-white crystalline powder was obtained.

1.5.3 Synthesis of ($Se_2P_2N_2$)$_2$·(DMU)·(DBTFB) (*T1*)

The milling jar was charged with a 10 mm diameter stainless steel ball bearing, **$Se_2P_2N_2$ (1)**, **DMU**, and **DBTFB** in a 2:2:1 ratio and the jar and sealed milled for 30 min. An off-white crystalline powder was obtained. Diffraction quality crystals were obtained from slow evaporation of Methanol under room temperature conditions from a mixture of 2:1:1 of **1**, **DMU**, and **DBTFB**.

1.5.4 Synthesis of ($Se_2P_2N_2$)·(18-crown-6 ether)$_2$·(KBr) (*T2*)

The milling jar was charged with a 10 mm diameter stainless steel ball bearing, **$Se_2P_2N_2$ (1)**, **18-crown-6 ether**, and **KBr** in a 2:1:1 ratio and the jar and sealed milled for 30 min. An off-white crystalline powder was obtained. Diffraction quality crystals were obtained from slow evaporation of $CHCl_3$ under room temperature conditions from a mixture of 1:2:1 of Compound **1**, **18-crown-6 ether**, and **KBr**.

1.5.5 Synthesis of ($Se_2P_2N_2$)$_2$·(18-crown-6 ether)$_2$·(KBr)·(DBTFB) (*Q*)

The milling jar was charged with a 10 mm diameter stainless steel ball bearing, **$Se_2P_2N_2$ (1)**, **18-crown-6 ether**, **DBTFB** and **KBr** in a 2:2:2:1 ratio and the jar and sealed milled for 30 min. An off-white crystalline powder was obtained. Diffraction quality crystals were obtained from slow evaporation of a mixture of THF and Ethyl Acetate 1:1 at room temperature using a mixture of 2:2:2:1 ratio of **1**, **18-crown-6 ether**, **DBTFB** and **KBr**.

1.5.6 Synthesis of ($Se_2P_2N_2$)$_2$·(18-crown-6 ether)$_2$·(KBr) ·(DBTFB) by combination of powders

The milling jar was charged with a 10 mm diameter stainless steel ball bearing, corresponding pre-obtained powders (Scheme 3) ratio and the jar and sealed milled for 30 min. Off-white crystalline powders were obtained.

1.6 Computational methods

1.6.1 Periodic DFT-D optimization of experimental crystal structures

The equilibrium geometry for each crystal structure (**B1, B2-Form I, B2-Form II, T1, T2** and **Q**) was computed using a periodic dispersion-corrected density functional theory (DFT-D) model as implemented in VASP,[1-3] which uses the projector-augmented wave (PAW)[4] method with plane-wave basis sets and PAW pseudo-potentials. In all cases, the Perdew-Burcke-Ernzerhof (PBE)[5] generalized gradient approximation (GGA) was used for the exchange-correlational functional coupled with the D3 dispersion-correction.[6] VASP input files were generated using the cif2cell[7] program. In order to aid rapid convergence, a two-stage geometry optimization protocol was employed: initially a fixed-cell geometry optimization was performed, after which the converged structure was used as input for a full geometry optimization of both the atom positions and cell parameters. In all cases, a Γ-centered Monkhorst-Pack scheme was used to generate a tight K-point mesh with maximum K-point distance set to $2\pi\times$ 0.032 Å^{-1}. In all calculations, the cut-off energy for the planewave basis was fixed at 500 eV. Within each self-consistent field cycle, the convergence threshold was set at 1×10^{-5} eV and the geometry was considered converged when all forces were below 0.03 eV Å^{-1}. The bulk cocrystal stabilization energy (ΔE_{coc}) for each cocrystal was estimated as the difference in the DFT-D energy of

the cocrystal relative to the stoichiometrically weighted sum of the energies of the reference single-component crystal structures. Where reference crystal structures for the components was not available, ΔE_{coc} could not be estimated. In all cases, the fully relaxed (atom positions and cell parameters) DFT-D energies of the crystal structures was used to compute ΔE_{coc}.

1.6.2 Complexation energy calculations

The resulting optimized structure following fixed-cell geometry optimization in VASP (stage 1) was used as the starting point for estimation of the thermodynamic driving force for complex formation (ΔE_{comp}). This was achieved by extracting the contents of the asymmetric unit for each VASP stage-1 optimized (see previous section for details) CIF and using GAUSSIAN09[8] to perform a single-point DFT-D energy evaluation at the B97D/6-31G(d,p) level of theory. Complexation energies were corrected for the basis set superposition error using the counterpoise method of Boys and Bernardi.[9]

1.6.3 Molecular electrostatic potential (MEP) surfaces

The molecular geometries for **1** (exo-exo and exo-endo conformations), **DMU** and **DBTFB** were optimized in the gas phase at the B97D/6-31G(d,p) level of theory using GAUSSIAN09. In order to define the correct geometry for the NH donors for each conformation of **1**, the dihedrals defining the relative orientation of the NH donors were constrained to their experimental values with all other degrees of freedom allowed to optimize freely. For the relatively rigid **DMU** and **DBTFB** molecules, no constraints were applied during the gas phase geometry optimization. Molecular electrostatic potentials (MEPs) were calculated at the B97D/6-31G(d,p) level of theory and visualized in GaussView 5.0.[10] Population analysis was performed using the Merz-Singh-Kollman scheme.[11-12] The atomic radius for Se was assumed to be 1.90 Å. In all cases, the equilibrium geometry for the isolated molecules were used as input for the MEP calculations. Local minima and maxima on the MEP surface (0.0004 au isodensity surface) were calculated using a positive point charge in vacuum as a probe. The calculations lead to the interaction energy (in kJ mol^{-1}) between the positive point probe and the surface of the molecule at the point of contact.

1.6.4 Mechanical properties of crystals

The VASP optimized (atom positions and lattice parameters) crystal structures for **B1**, **B2** (Forms I and II) and **T1** were loaded into BIOVIA Materials Studio 8.0.[13] Crystals **T2** and **Q** contain atom types with no available force fields in Materials Studio, thus the properties of these crystals were not modelled. The Forcite module of BIOVIA Materials Studio 8.0 was used to perform an initial geometry optimization (atom positions and lattice parameters) using the Dreiding[14] force field supplemented with atomic charges obtained using the charge equilibration (QEq) method.[15] The convergence thresholds used were an energy of 1×10^{-3} kcal mol^{-1}, maximum force of 0.5 kcal mol^{-1} Å^{-1}, maximum stress of 0.5 GPa

and maximum displacement of 1.5×10^{-2} Å. Following the geometry optimization, Forcite was used once again to compute the mechanical properties of the optimized crystal structures. This was achieved using the same energy model and same convergence thresholds specified above.

1.6.5 Monte Carlo binding geometries

The gas-phase optimized molecular structures of **1**, **DBTFB** and **DMU** were derived at the B97D/6-31G(d,p) level of theory using GAUSSIAN09. The atomic charges for the resulting conformational energy minimum was computed using the Dmol^3 module of BIOVIA Materials Studio 8.0. In all cases, the generalized gradient approximation (GGA) and PBE functional were used. The calculations assume the DNP basis set and Grimme's G06[9] dispersion correction. The convergence thresholds used were an energy threshold of 2×10^{-5} Hartree, a maximum force of 4×10^{-3} Hartree Å^{-1} and a maximum displacement of 5×10^{-3} Å. Favorable binding geometries between **1** and **DBTFB**/**DMU** were calculated using the Blends module of BIOVIA Materials Studio 8.0 which uses a weighted Monte Carlo sampling algorithm[20] to map the configurational space for binding between sets of input molecular structures. For each search for stable binary complexes between **1** and **DBTFB**/**DMU**, a total of 100,000 pair configurations were generated during the Monte Carlo search. The stability of each pair configuration was evaluated using the Dreiding force field[10] complemented with the Dmol^3 derived atomic charges. The 100 most stable configurations following each Monte Carlo search were retained for visualizing the binding modes between **1** and coformer molecules.

1.6.6 Crystal density, packing index and void volume calculations

Using the experimental crystal structures as input, PLATON[16] was used to estimate the crystal density, packing index (PI) and % solvent accessible void volume (V_{void}). For calculations of V_{void} for each crystal structure, the default probe radius of 1.2 Å was used.

2. Powder X-Ray Diffraction (PXRD) Patterns

For each route of successful formation of a binary, ternary or quaternary cocrystal, the obtained PXRD pattern is stacked against its starting materials to show an absence thereof, as well as against its simulated pattern(s) obtained from the single crystal diffraction data to show a match.

For each route of unsuccessful formation of a binary, ternary or quaternary cocrystal, the obtained PXRD pattern is stacked against its starting material to show their presence and/or against the pattern of the suspected product(s).

A simple list of all the figures is shown below:

2.1 Binary cocrystals

S1. (**$Se_2P_2N_2$**)·(**DBTFB**) – route B1

S2. (**$Se_2P_2N_2$**)·(**DMU**) – route B2

S3. (**$Se_2P_2N_2$**) + (**KBr**) – cocrystal not formed

2.2 Ternary cocrystals

S4. (**$Se_2P_2N_2$**)·(**DBTFB**)·(**DMU**) – route $T1_1$

S5. (**$Se_2P_2N_2$**)·(**DBTFB**)·(**DMU**) – route $T1_2$

S6. (**$Se_2P_2N_2$**)·(**18-crown-6 ether**)·(**KBr**) – route T2

2.3 Quaternary cocrystals (Se₂P₂N₂) (DBTFB)·(18-crown-6 ether)·(KBr)

S7. Route Q_1

S8. Route Q_2

S9. Route Q_3

S10. Route Q_4 – cocrystal not formed

S11 Route Q_5 – cocrystal not formed

2.1 Binary cocrystals

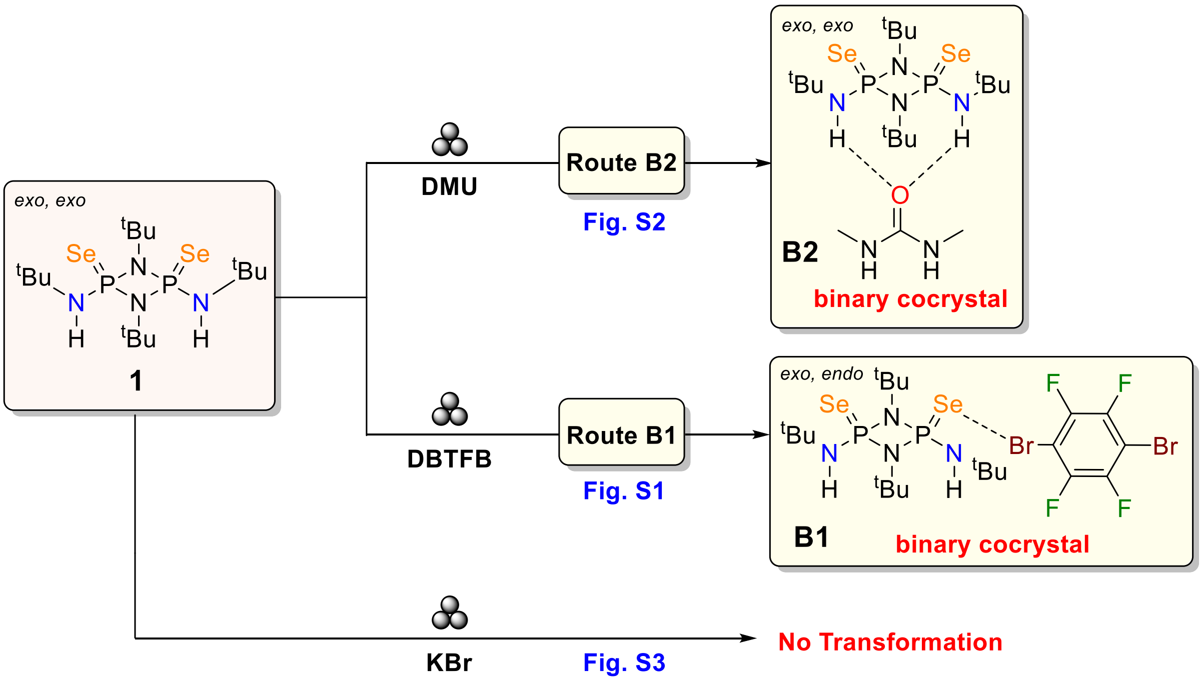

Scheme S1. Synthetic pathways to obtain binary cocrystals, **B1** and **B2** from **1**.

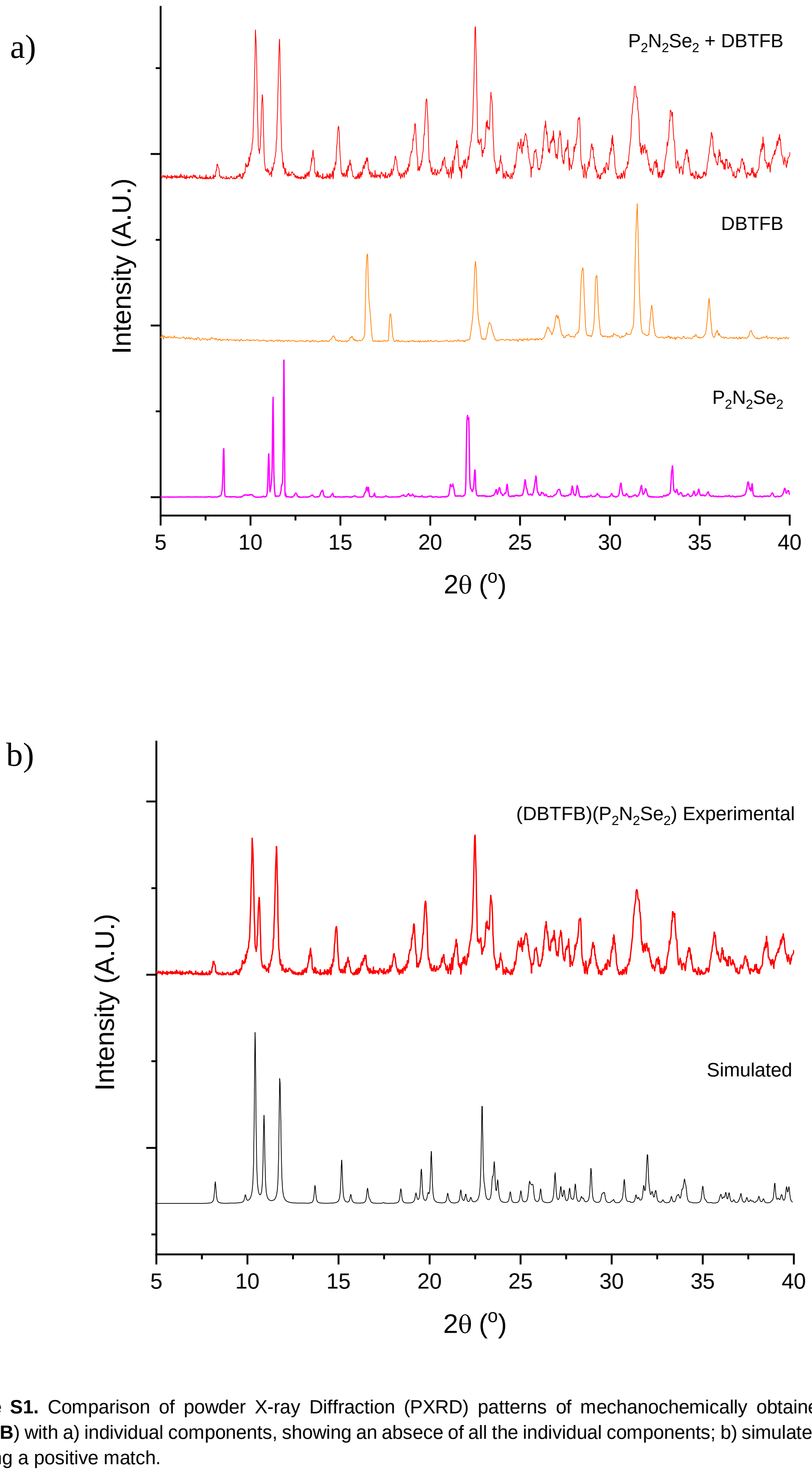

Figure S1. Comparison of powder X-ray Diffraction (PXRD) patterns of mechanochemically obtained powder of (**$Se_2P_2N_2$**) (**DBTFB**) with a) individual components, showing an absece of all the individual components; b) simulated patterns from SC-XRD showing a positive match.

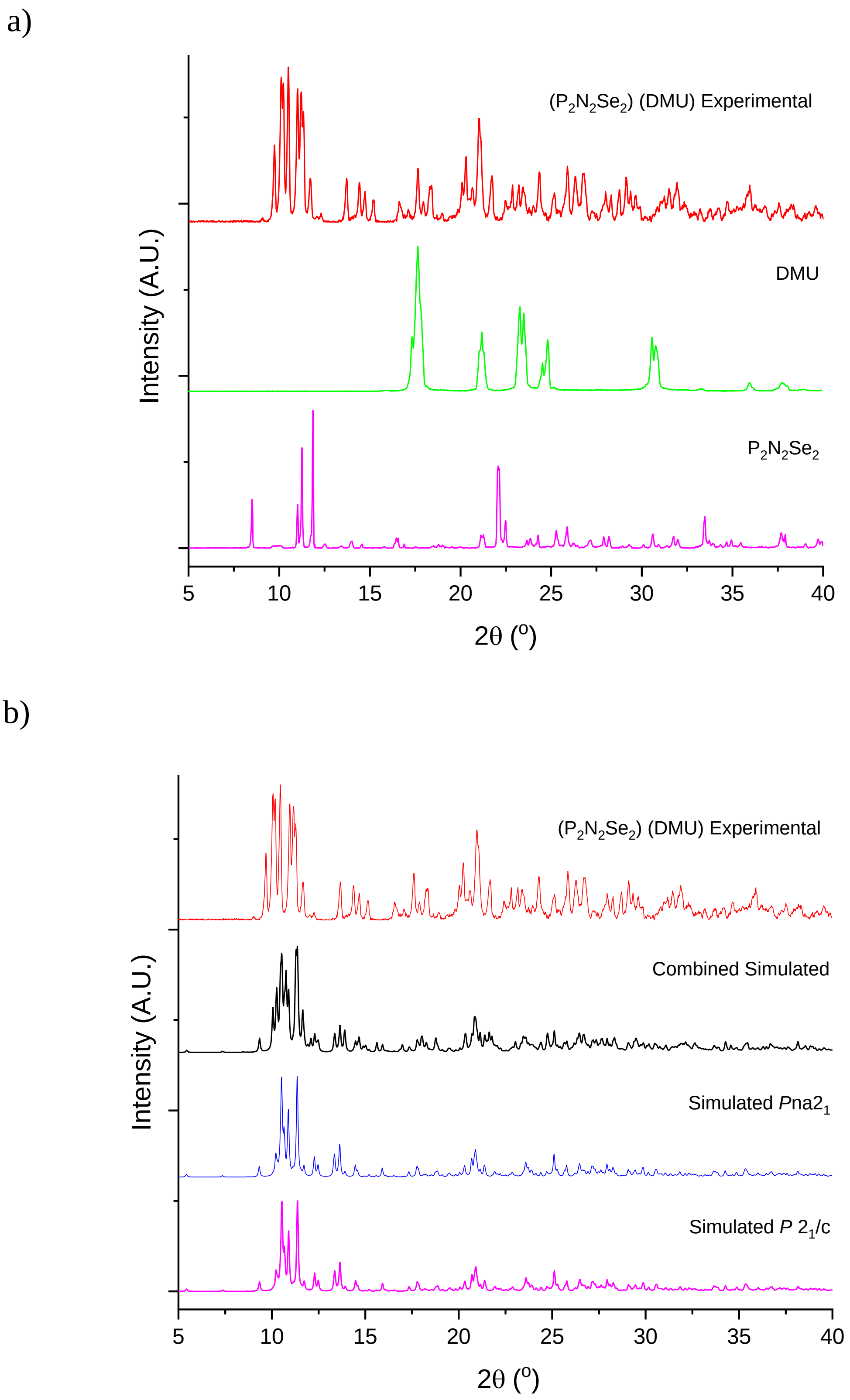

Figure S2. Comparison of powder X-ray Diffraction (PXRD) patterns of mechanochemically obtained powder of (**$Se_2P_2N_2$**) (**DMU**) with a) individual components, showing an absece of all the individual components; b) simulated patterns from SC-XRD showing a positive match.

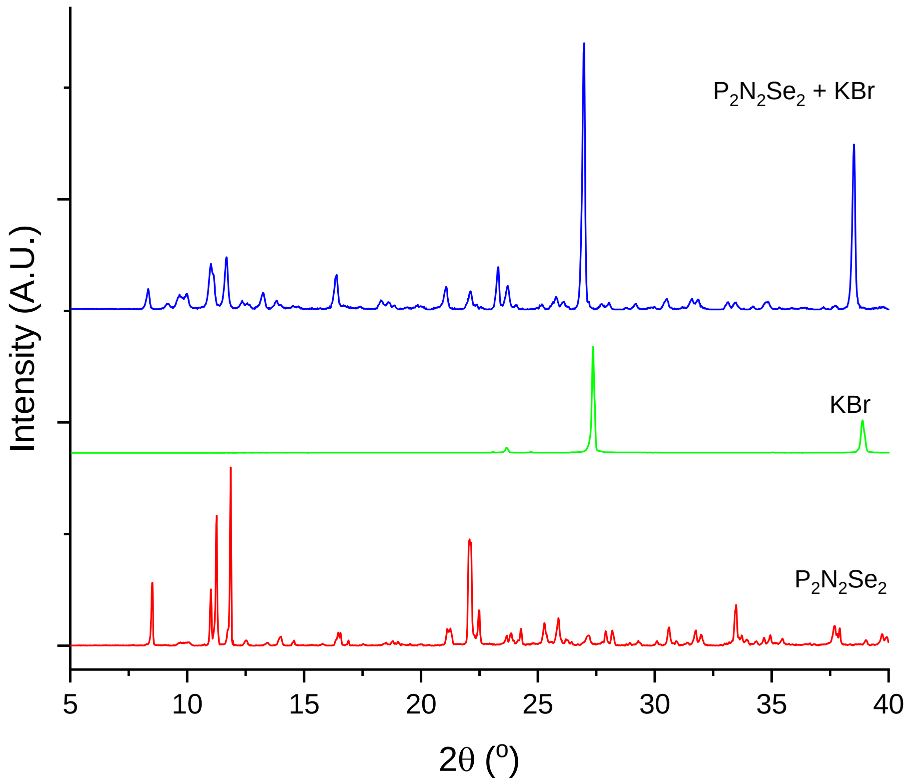

Figure S3. Comparison of powder X-ray Diffraction (PXRD) patterns of mechanochemically obtained powder of (**$Se_2P_2N_2$**)+(**KBr**) with the individual components, indicating a powder mixture of the starting materials.

2.2 Ternary cocrystals

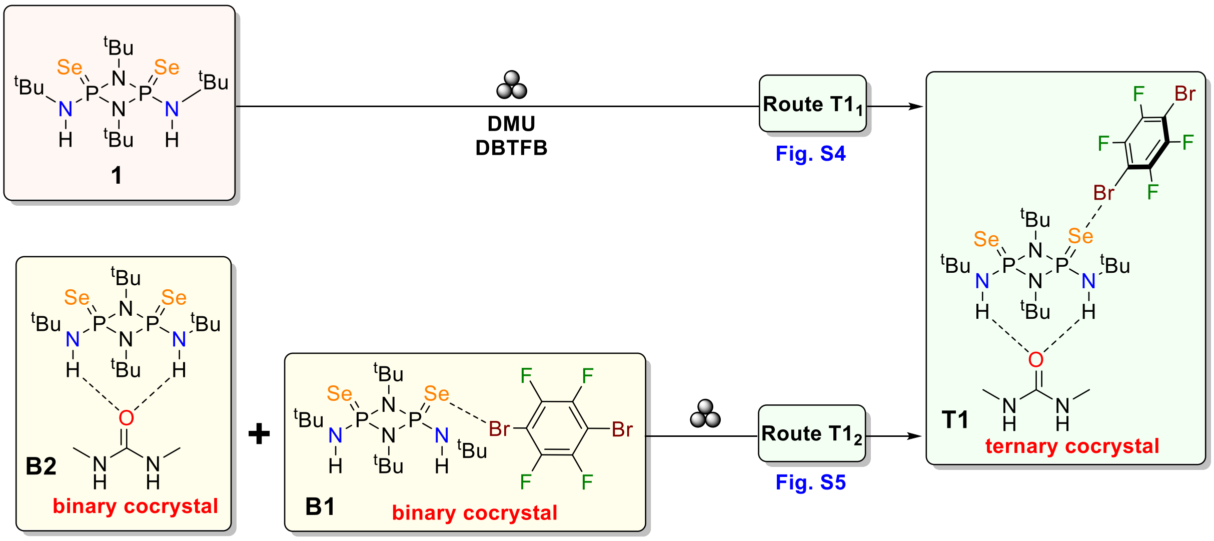

Scheme S2. Synthetic pathways to obtain ternary cocrystal, **T1.**

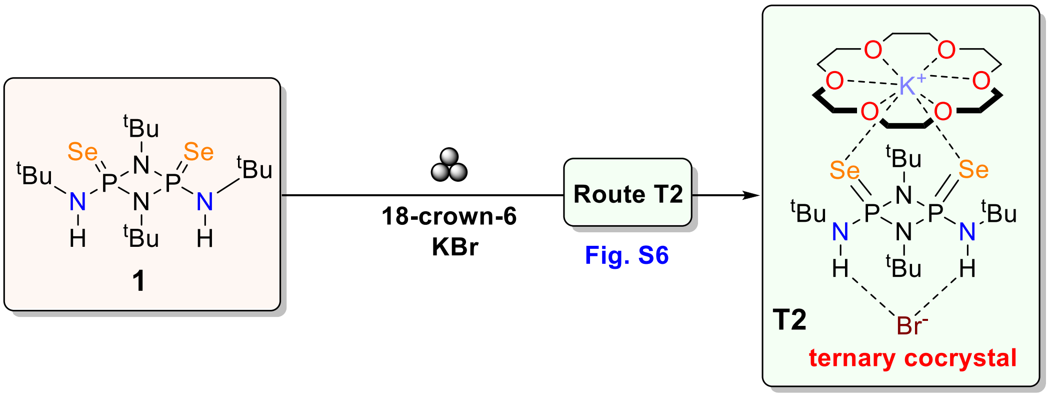

Scheme S3. Synthetic pathways to obtain ternary cocrystal, **T2**.

a)

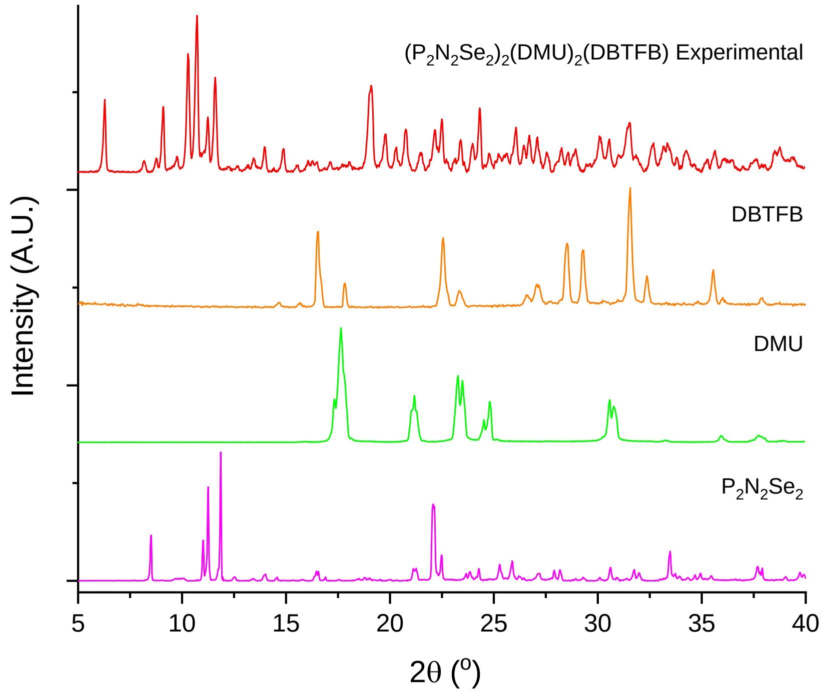

b)

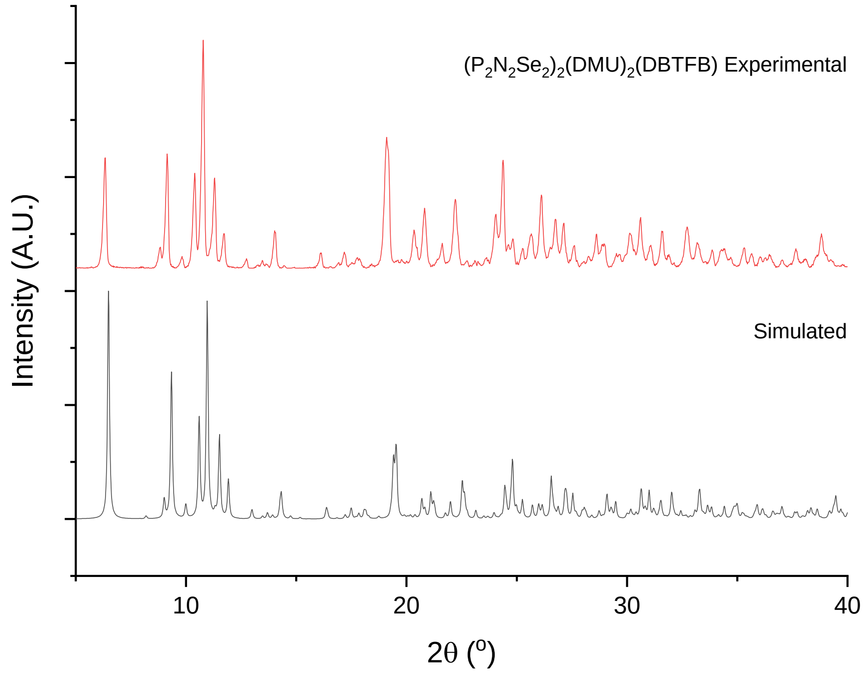

Figure S4. Comparison of powder X-ray Diffraction (PXRD) patterns of mechanochemically obtained powder of (**$Se_2P_2N_2$**)(**DMU**)(**DBTFB**) in a 2:2:1 ratio with a) individual components, showing an absece of all the individual components; b) simulated patterns from SC-XRD showing a positive match.

a)

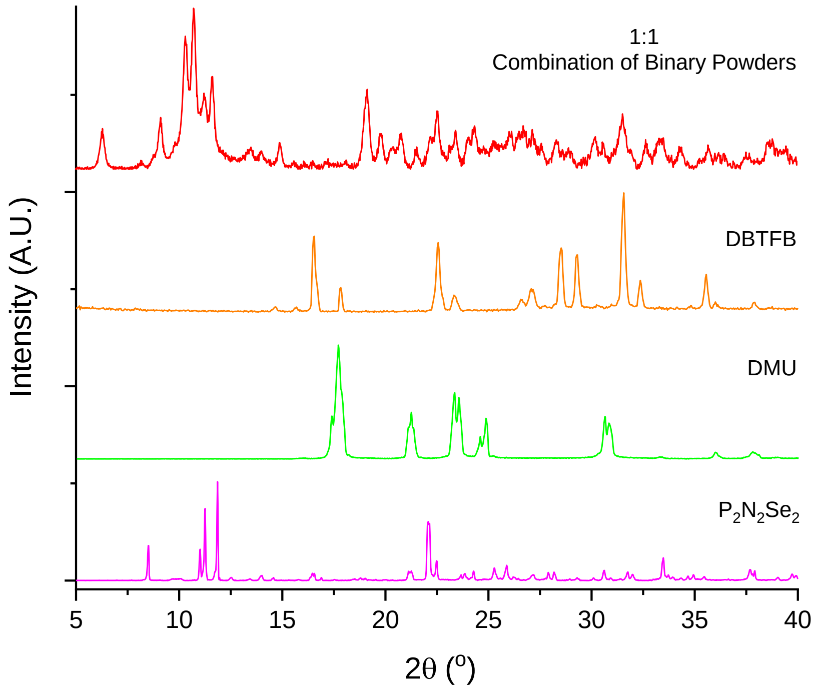

b)

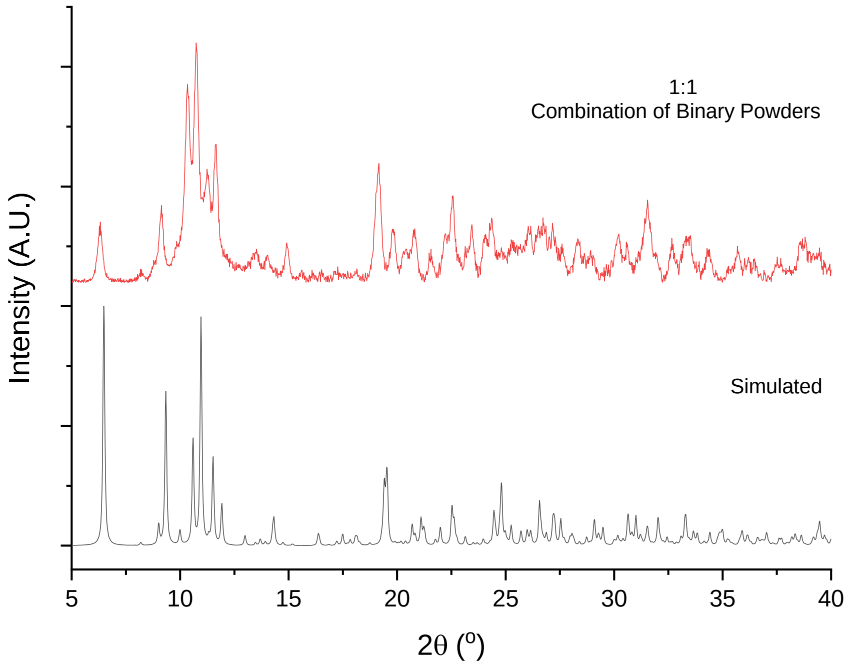

Figure S5. Comparison of powder X-ray Diffraction (PXRD) patterns of mechanochemically obtained ternary powder of (**$Se_2P_2N_2$**)(**DMU**)(**DBTFB**) from the combination of (**$Se_2P_2N_2$**)(**DMU**) and (**$Se_2P_2N_2$**)(**DBTFB**) in a 1:1 ratio, with a) individual components, showing an absece of all the individual components; b) simulated patterns from SC-XRD showing a positive match.

a)

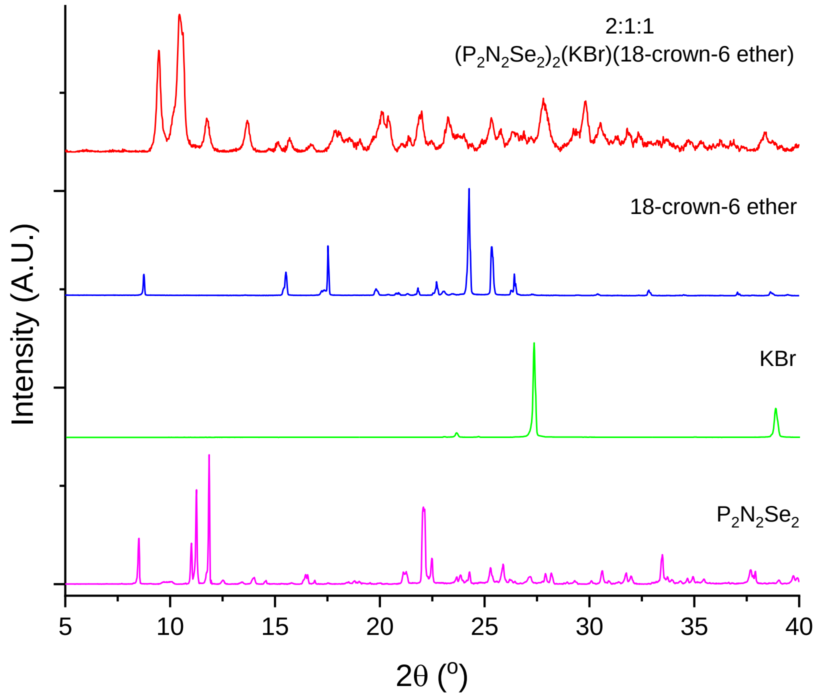

b)

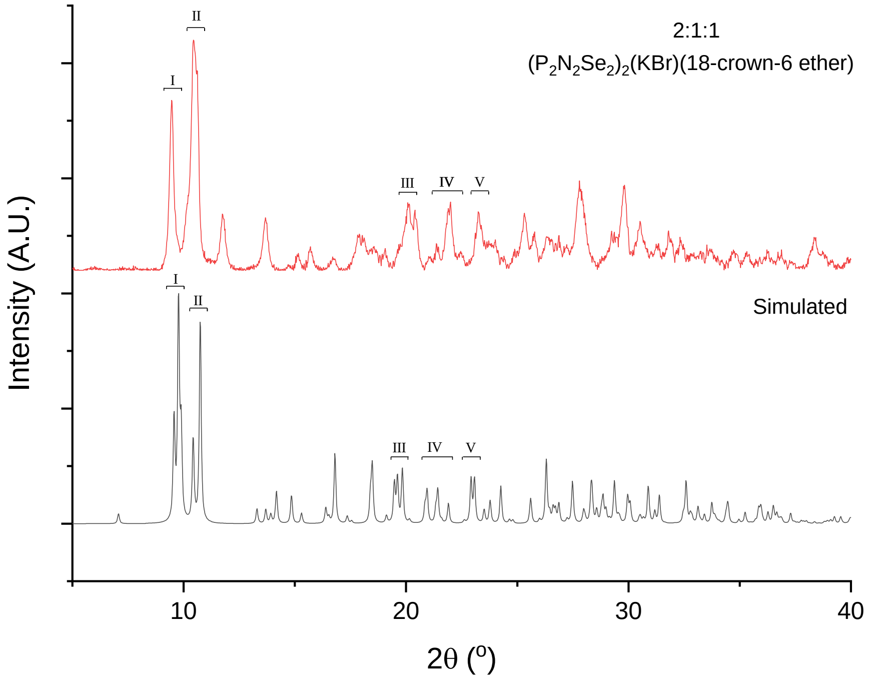

Figure S6. Comparison of powder X-ray Diffraction (PXRD) patterns of mechanochemically obtained powder of (**$Se_2P_2N_2$**)(**KBr**)(**18-crown-6 ether**) in a 2:1:1 ratio with a) individual components, showing an absece of all the individual components; b) simulated patterns from SC-XRD showing a positive match.

2.3 Quaternary cocrystals

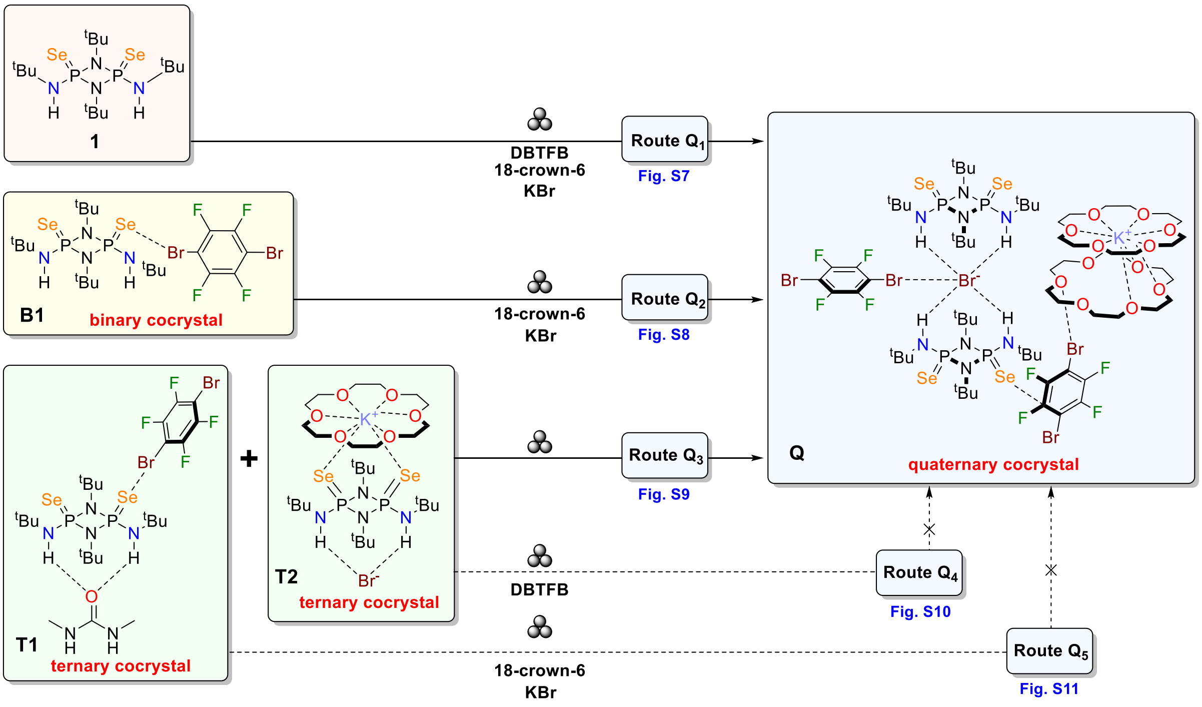

Scheme S4. Synthetic pathways to obtain quaternary cocrystal, **Q**. Routes **Q_1**, **Q_2**, and **Q_3** denotes the successful syntheses; **1** and **B1** was combined with the respective starting materials whereas **T1** and **T2** were combined to afford **Q**. Combination of **T1** and **T2** with the respective starting materials could not yield **Q**.

a)

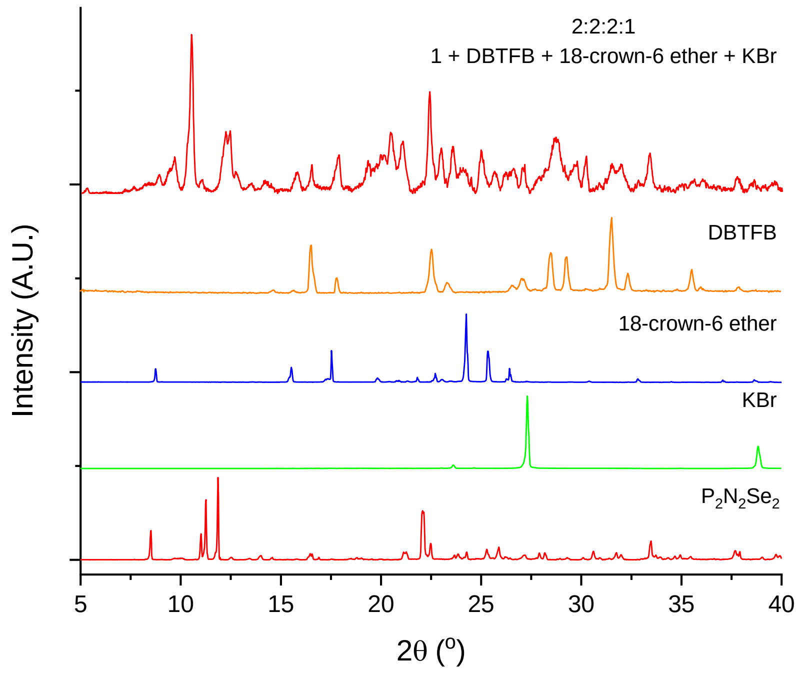

b)

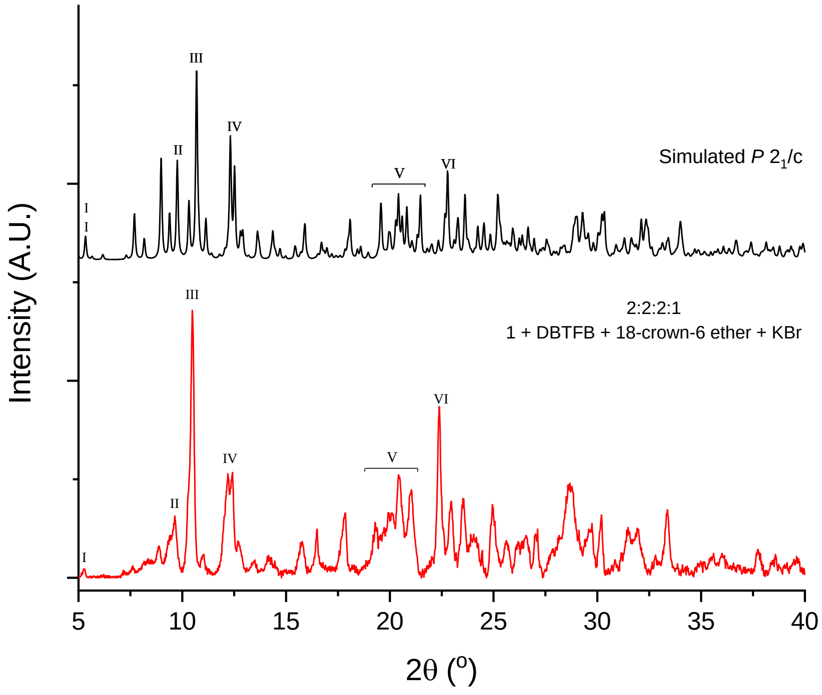

Figure S7. Comparison of powder X-ray Diffraction (PXRD) patterns of mechanochemically obtained powder of (**$Se_2P_2N_2$**)(**DBTFB**)·(**18-crown-6 ether**)·(**KBr**) with a) individual components, the patterns show an absece of all the individual components; b) simulated patterns from SC-XRD, *Roman Numerals* denote the matching peaks, indicating that the bulk of the powder obtained corresponds to the quaternary cocrystal, **Q**.

a)

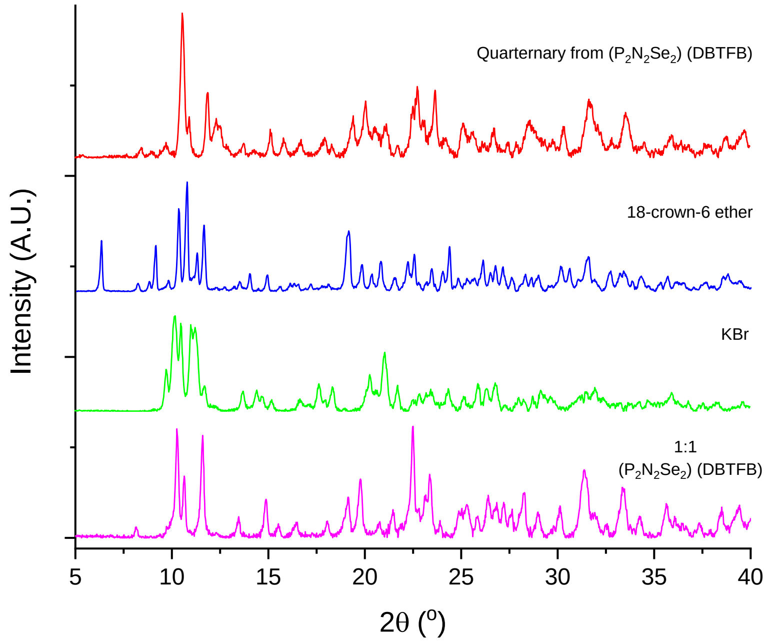

b)

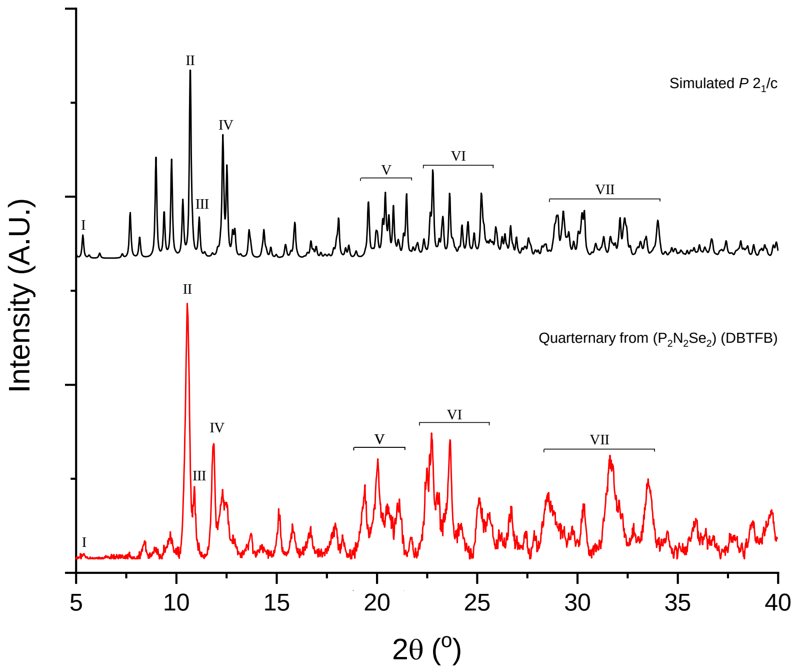

Figure S8. Comparison of powder X-ray Diffraction (PXRD) patterns of mechanochemically mechanochemical combination of obtained binary powder of (**$Se_2P_2N_2$**)·(**DBTFB**) with **18-crown-6** ether and **KBr** and a) all obtained binary and ternary powders as labelled. As the starting matrial of this route is of a binary cocrystal, the comparison of all the other binary and ternary powders show an absence of them, hence a possible conversion to our predicted quarternary product; b) simulated pattern from SC-XRD, *Roman Numerals* denote the matching peaks obtained corresponds to the quaternary cocrystal, **Q**.

a)

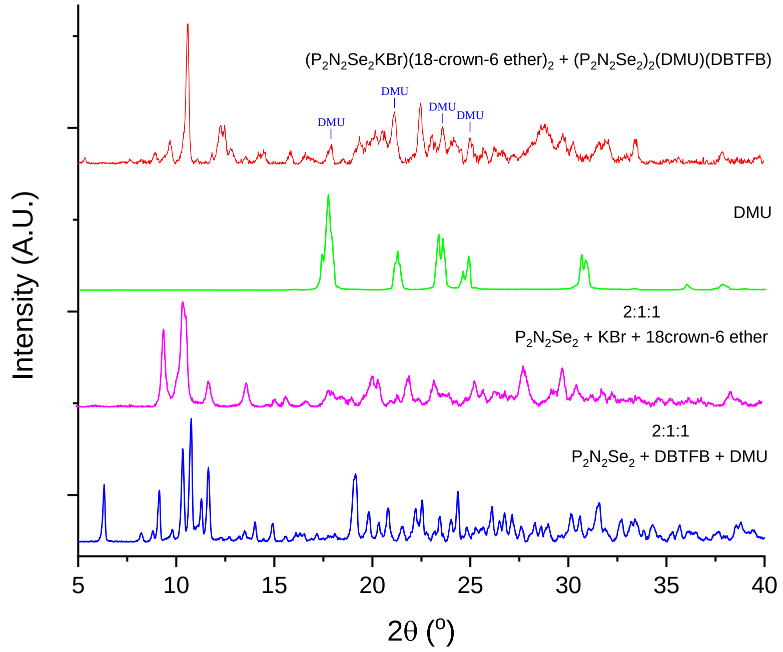

b)

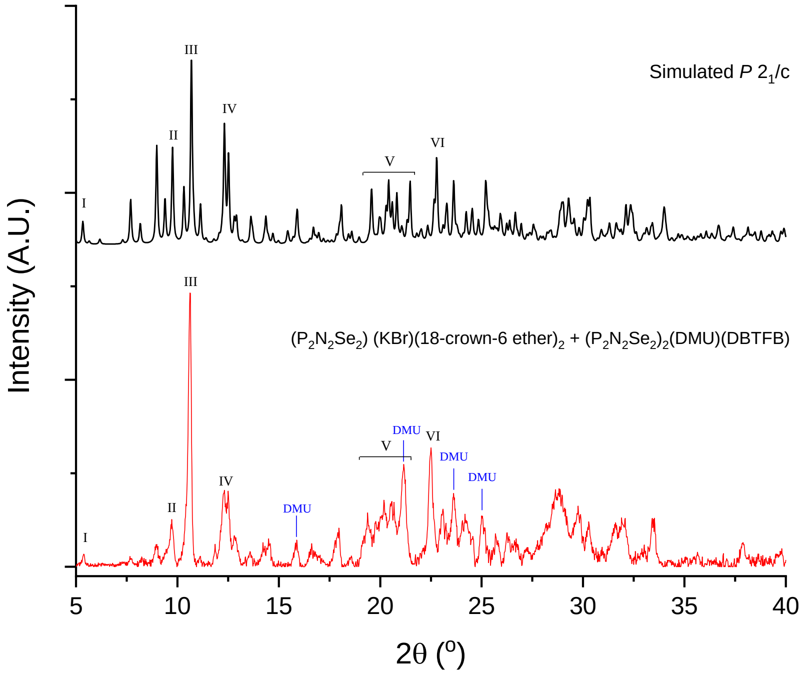

Figure S9. Comparison of powder X-ray Diffraction (PXRD) patterns of mechanochemical combination of both obtained ternary powder of (**$Se_2P_2N_2$**)·(**DBTFB**)·(**DMU**) with (**$Se_2P_2N_2$**)·(**18-crown-6 ether**)·(**KBr**) and a) all obtained binary and ternary powders as labelled. As the starting matrial of this route is of a binary cocrystal, the comparison of all the other binary and ternary powders show an absence of them, hence a possible conversion to our predicted quarternary product; b) simulated pattern from SC-XRD, *Roman Numerals* denote the matching peaks, indicating that the bulk of the powder obtained obtained corresponds to the quaternary cocrystal, **Q**. The **DMU** which from the ternary cocrystal **T1**, is believed to be displaced and exists in the final powder mixture as itself; corresponding peaks have been labelled.

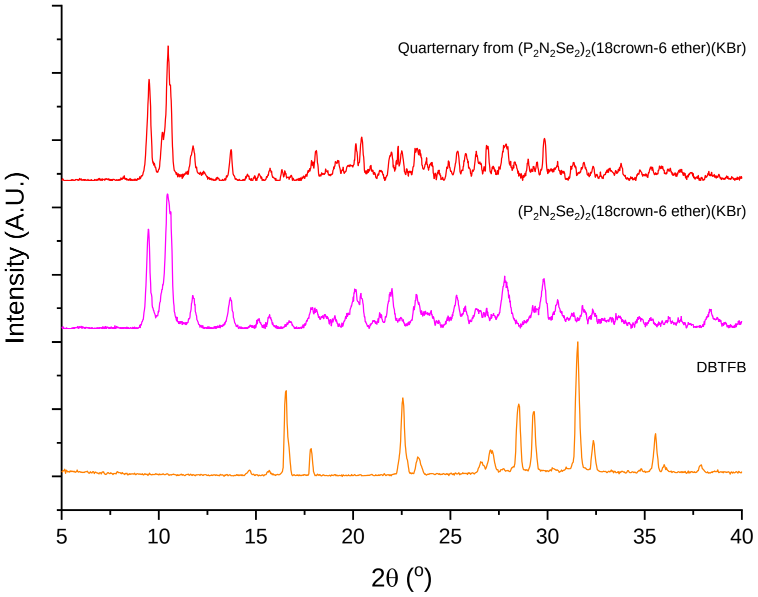

Figure S10. Comparison of powder X-ray Diffraction (PXRD) patterns of mechanochemical combination of obtained Ternary powder of (**$Se_2P_2N_2$**)·(**18-crown-6 ether**)·(**KBr**) with **DBTFB** and obtained binary and ternary powders as labelled; *Roman Numerals* denote the matching peaks. Matching peaks indicated that an attempted formation of the quaternary cocrystals using the stated ternary powder did not work, instead, a mixture of the initial ternary cocrystals and **DBTFB** was formed.

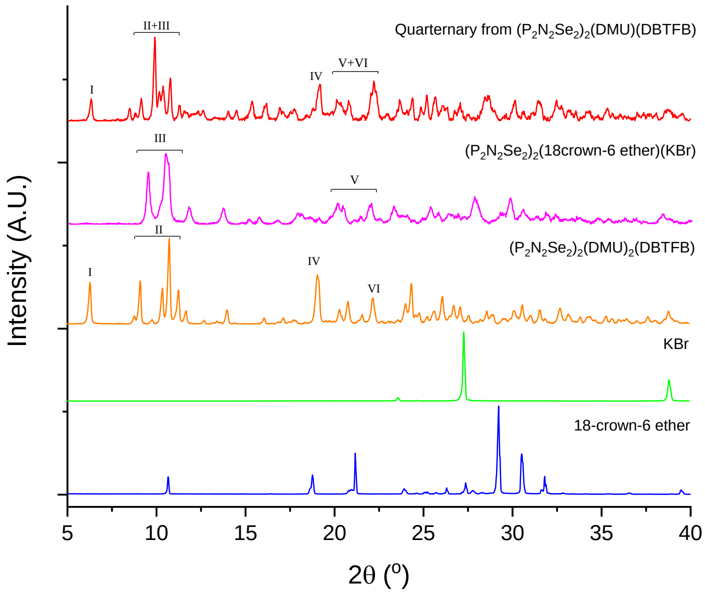

Figure S11. Comparison of powder X-ray Diffraction (PXRD) patterns of mechanochemical combination of obtained Ternary powder of (**$Se_2P_2N_2$**)·(**DBTFB**)·(**DMU**) with **18-crown-6** ether and **KBr** and obtained binary and ternary powders as labelled; *Roman Numerals* denote the matching peaks. Matching peaks indicated that an attempted formation of the quaternary cocrystals using the stated ternary powder did not work, instead, a mixture of both the ternary cocrystals are formed.

3. Spectroscopic Data for Crystals

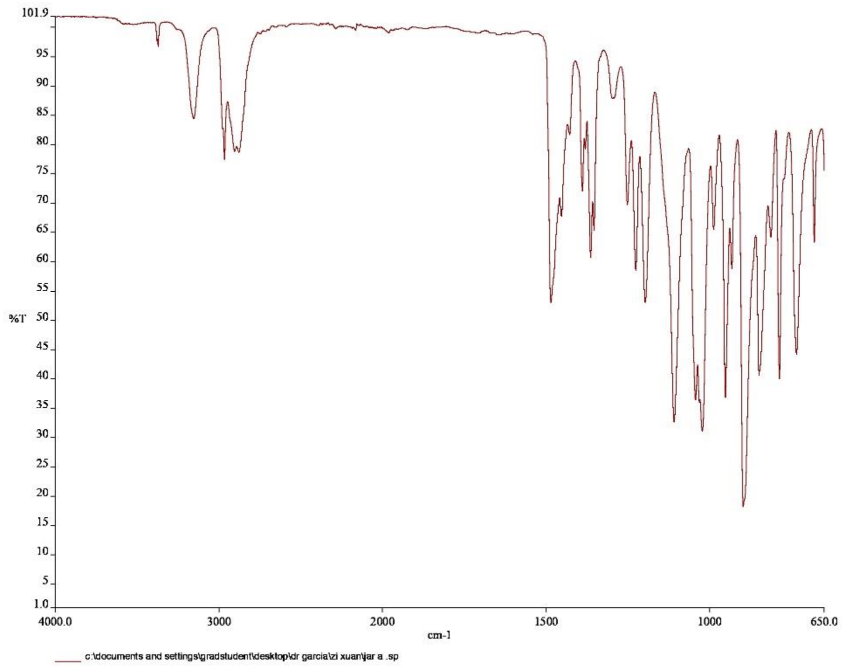

Figure S12. AT-IR Spectrum of mechanochemically mechanochemical combination of obtained binary powder of (**$Se_2P_2N_2$**)·(**DBTFB**) with **18-crown-6 ether** and **KBr.**

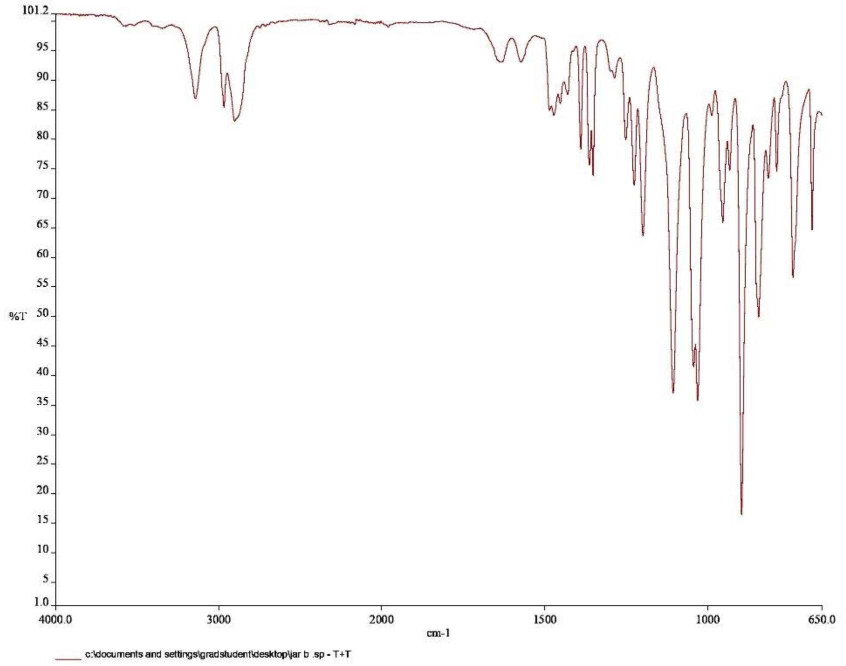

Figure S13. AT-IR Spectrum of of mechanochemically obtained powder of a combination of (**$Se_2P_2N_2$**)·(**DBTFB**)·(**DMU**) with (**$Se_2P_2N_2$**)·(**18-crown-6 ether**)·(**KBr**).

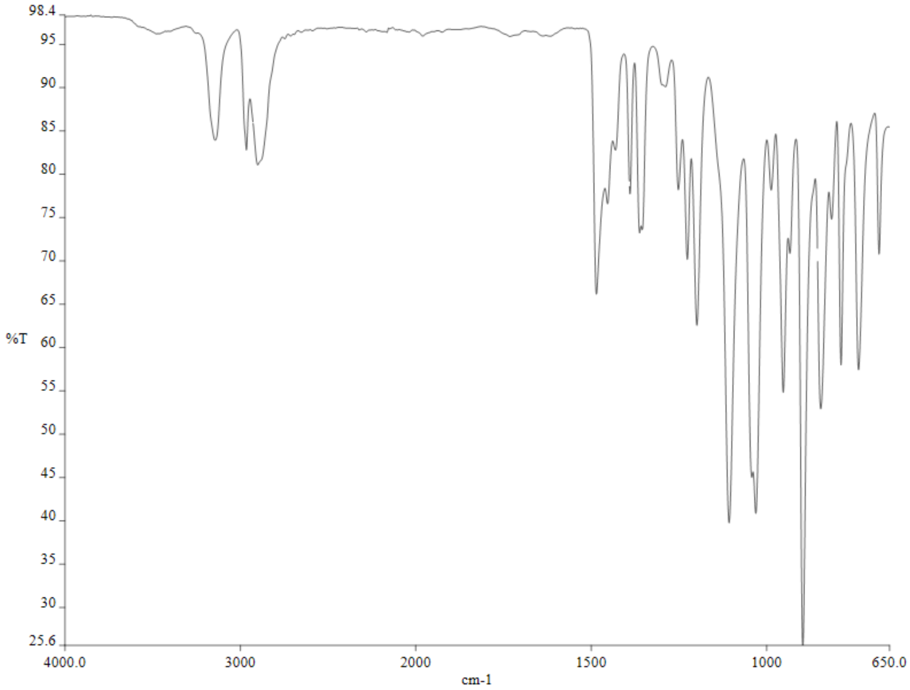

Figure S14. AT-IR Spectrum of mechanochemically obtained powder of (**$Se_2P_2N_2$**) (**DBTFB**)·(**18-crown-6 ether**)·(**KBr**).

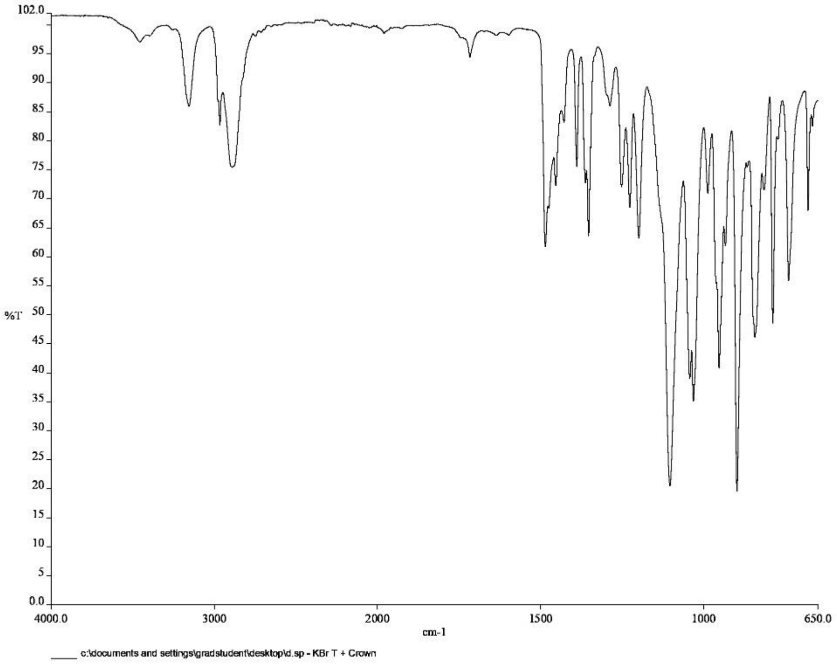

Figure S15. AT-IR Spectrum of mechanochemically mechanochemical combination of obtained Ternary powder of (**$Se_2P_2N_2$**)·(**18-crown-6 ether**)·(**KBr**) with **DBTFB**.

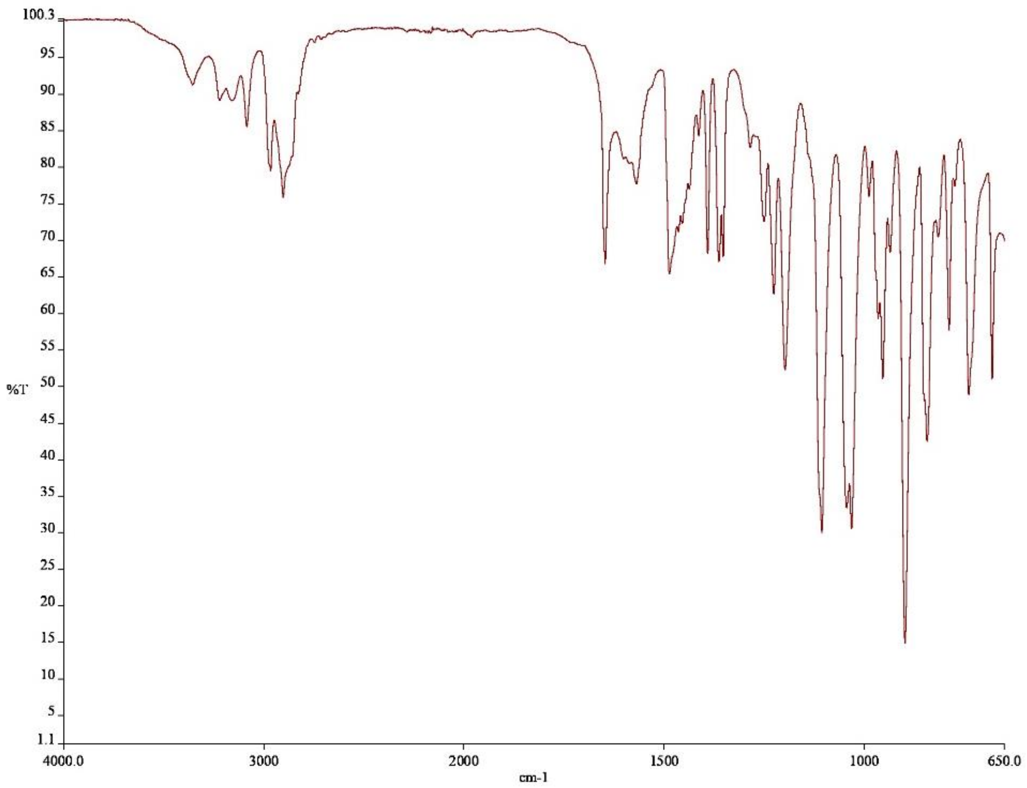

Figure S16. AT-IR Spectrum of mechanochemically mechanochemical combination of obtained Ternary powder of (**$Se_2P_2N_2$**)·(**DBTFB**)·(**DMU**) with **18-crown-6** ether and **KBr.**

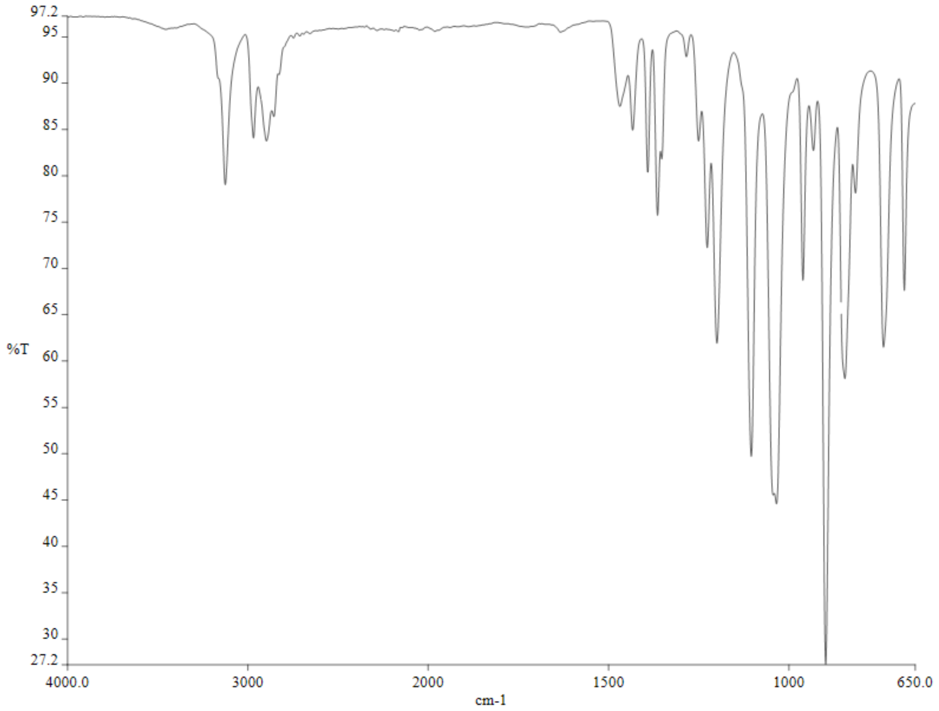

Figure S17. AT-IR Spectrum of mechanochemically obtained powder of (**$Se_2P_2N_2$**)·(**18-crown-6 ether**)·(**KBr**).

.

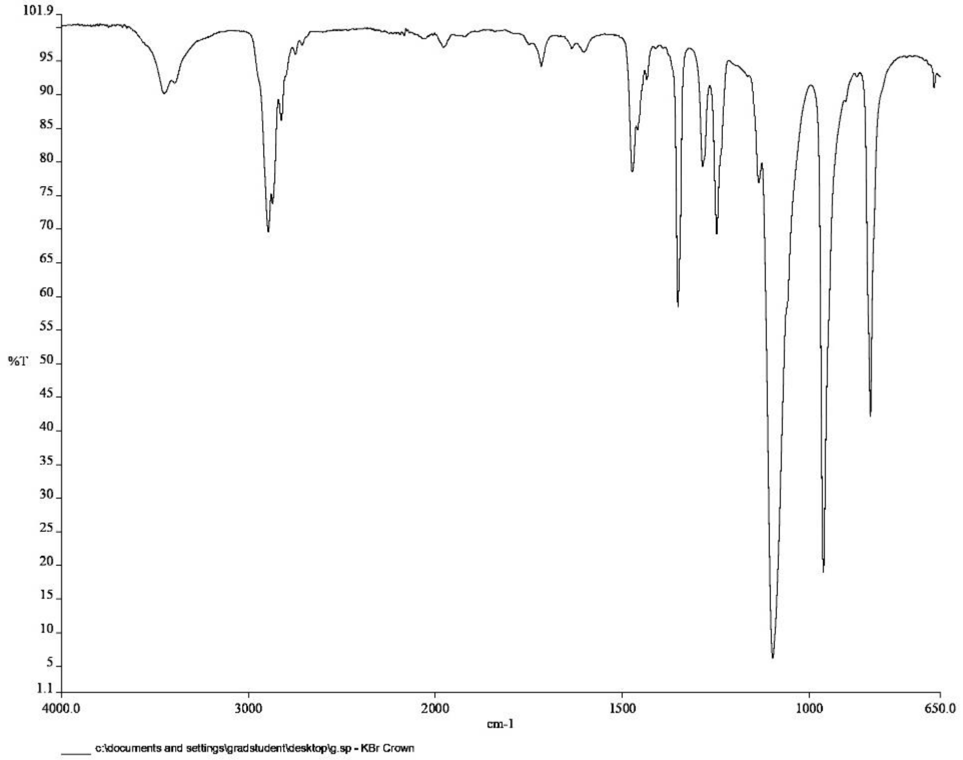

Figure S18. AT-IR Spectrum of mechanochemically obtained powder of (**$Se_2P_2N_2$** + **KBr**).

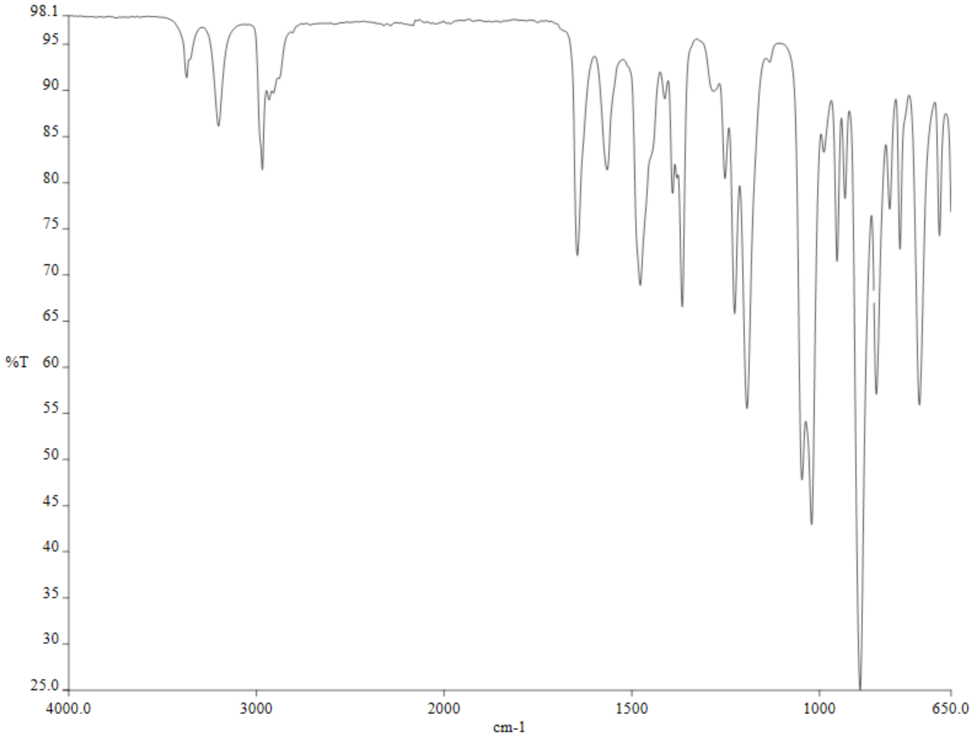

Figure S19. AT-IR Spectrum of mechanochemically obtained powder of ternary cocrystal of (**$Se_2P_2N_2$**)$_2$·(**DMU**)·(**DBTFB**) from its individual components in a 2:2:1 ratio.

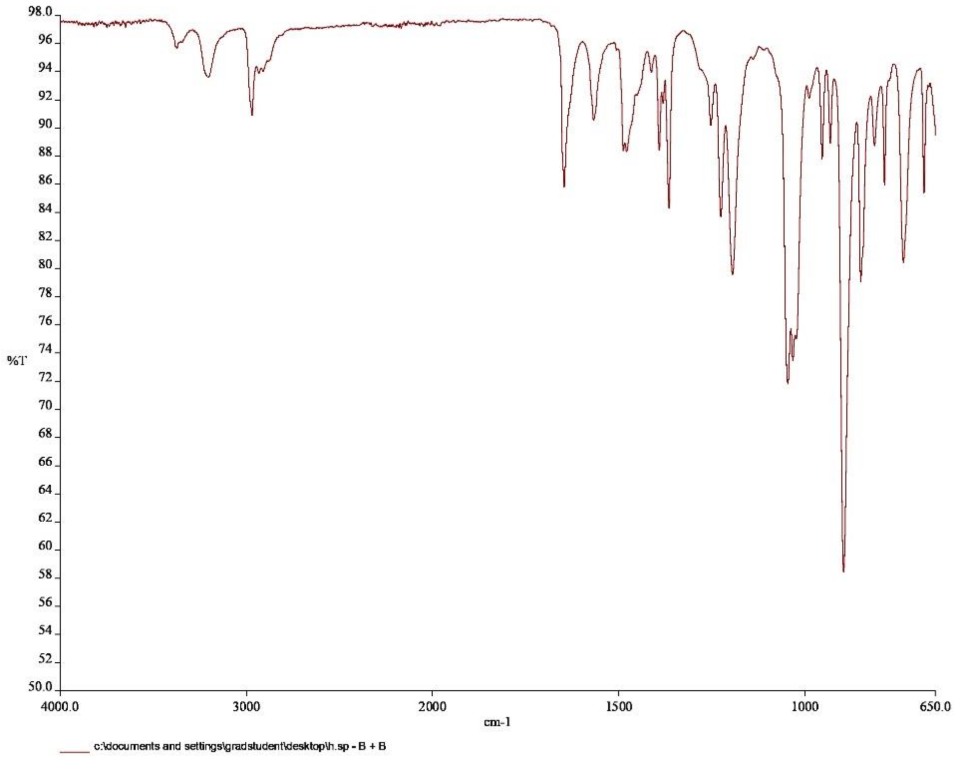

Figure S20. AT-IR Spectrum of mechanochemical combination of obtained binary powders of (**$Se_2P_2N_2$**)·(**DMU**) and (**$Se_2P_2N_2$**)·(**DBTFB**), yielding (**$Se_2P_2N_2$**)$_2$·(**DMU**)·(**DBTFB**).

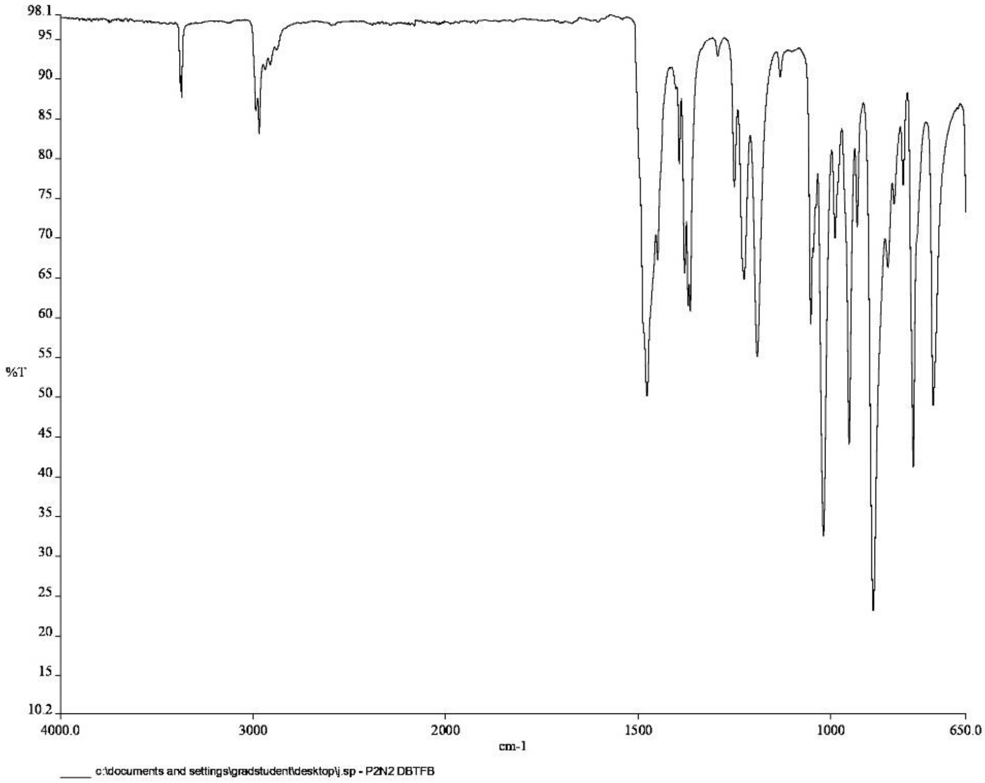

Figure S21. AT-IR Spectrum of (**$Se_2P_2N_2$**)·(**DBTFB**).

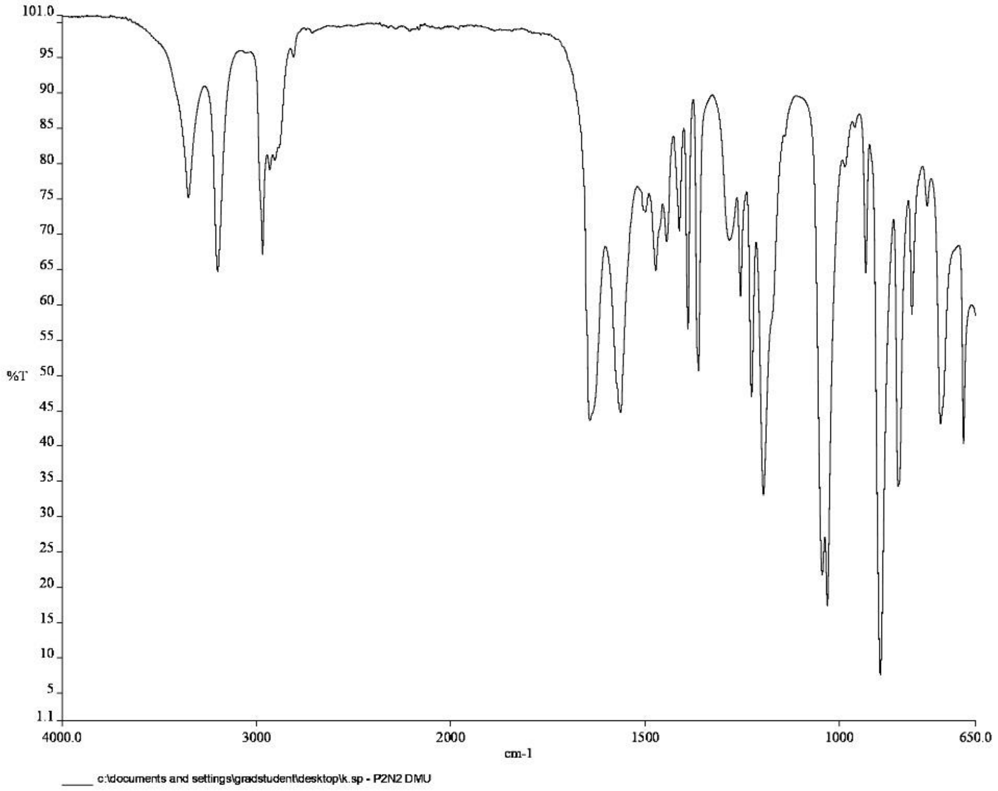

Figure S22. AT-IR Spectrum of (**$Se_2P_2N_2$**)·(**DMU**).

4. X-ray data of crystals 1-4

Crystal No.	T1	T2	Q
Empirical formula	$C_{22}H_{46}BrF_2N_6OP_2Se_2$	$C_{44}H_{100}BrKN_8O_6P_4Se_4$	$C_{68}H_{124}Br_5F_8KN_8O_{12}P_4Se_4$
Formula weight / gmol^{-1}	748.42	1396.04	2276.11
Crystal system	orthorhombic	Monoclinic	Monoclinic
Space Group	*P b c n*	*C* 1 2/*c*	*P* 1 2_1 / *c*
a/ Å	19.6129(4)	19.1871(7)	17.7708(5)
b/ Å	18.9223(3)	18.4670(7)	33.0772(10)
c/ Å	17.6931(4)	20.4771(10)	15.8981(4)
α/ °	90	90	90
β/ °	90	118	93.8745(18)
γ/ °	90	90	90
Volume/ Å^3	6566.3(2)	6409.9(5)	9323.7(5)
Density (calculated)/ gcm^{-3}	1.514	1.447	1.621
Absorp. Coeff./ mm^{-1}	3.603	3.123	6.055
F(000)	3032	2872	4584

Crystal size/ mm^3	0.240 x 0.360 x 0.400	0.100 x 0.140 x 0.200	0.020 x 0.200 x 0.220
θ range/ °	2.30 to 31.52	2.40 to 31.14	2.49 to 68.79
Index range	-27 ≤ h ≤ 28<br>-25 ≤ k ≤ 27<br>-26 ≤ l ≤ 25	-27 ≤ h ≤ 27,<br>-26 ≤ k ≤ 26,<br>-29 ≤ l ≤ 29	-21 ≤ h ≤ 21,<br>-39 ≤ k ≤ 37,<br>-19 ≤ l ≤ 19
Refl. Collected	65038	73904	113031
Indeo. Refns. (Rint)	10864 (0.0720)	10276 (0.0448)	17151 (0.0960)
Absop. Corr.	Multi-Scan	Multi-Scan	Multi-Scan
Max., Min., transmission	0.4780, 0.3270	0.7450, 0.5740	0.8880 and 0.3490
Refinement Method	Full-matrix least-squares on F^2	Full-matrix least-squares on F^2	Full-matrix least-squares on F^2
Data/restraint/parameters	10864 / 3 / 370	10276 / 714 / 406	17151 / 0 / 1009
Goodness-of-fit on F2	1.929	1.053	1.213
Final R indices [I>2σ(I)]	R1=0.0358, wR2 = 0.0584	R1 = 0.0405, wR2 = 0.1055	R1 = 0.0764, wR2 = 0.1807
R indices (all data)	R1 = 0.0695, wR2 = 0.0668	R1 = 0.0537, wR2 = 0.1134	R1 = 0.0896, wR2 = 0.1873
Largest diff. peak and hole/ $eÅ^{-3}$	0.479 and -0.526	1.978 and -1.168	1.519 and -1.227

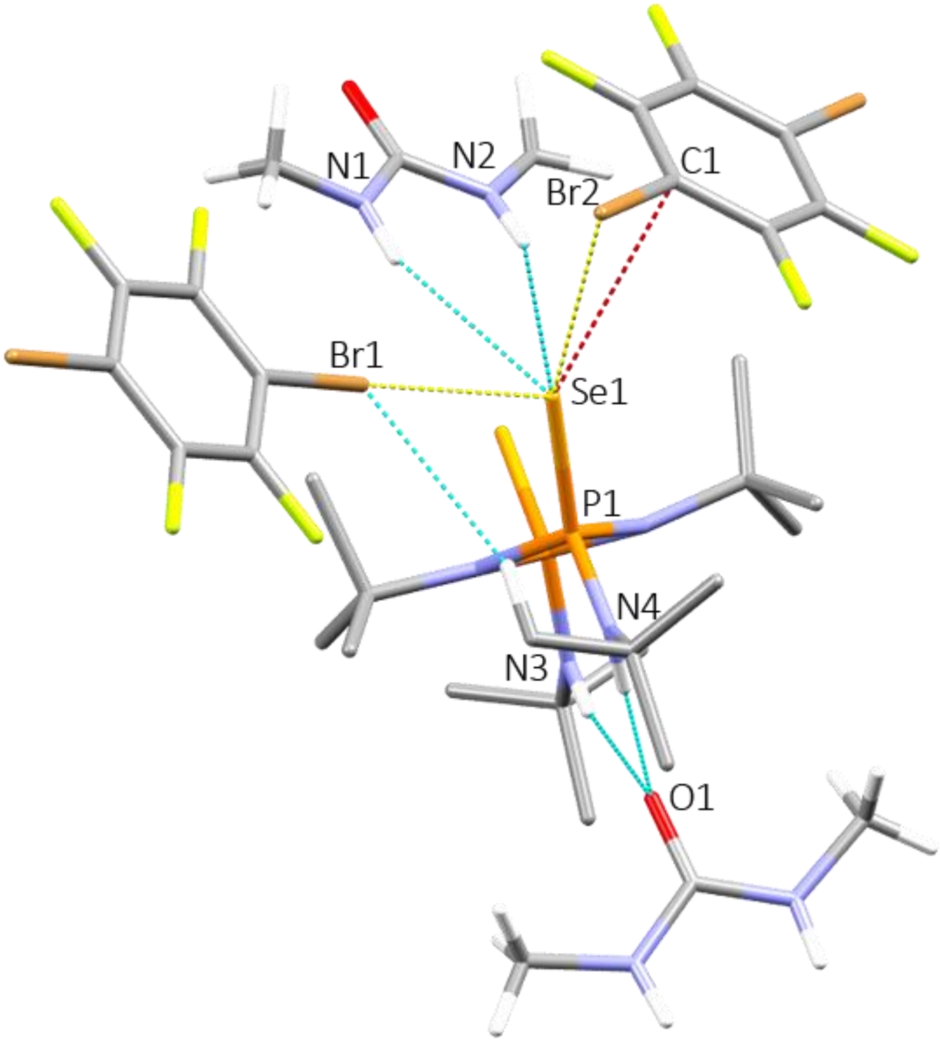

Figure S22: Crystal 1 (**$Se_2P_2N_2$**)·(**DBTFB**)·(**DMU**); Selected lengths of intermolecular interactions [Å] and Angles [°]: Se(1)···N(1) 3.712, Se(1)···N(2) 3.780, Se(1)···Br(1) 3.422, Se(1)···Br(2) 3.747, Se(1)···C(1) 3.453, O(1)···N(3) 2.928, O(1)···N(4) 2.982; N(1)···Se(1)···N(2) 35.84, N(3)···O(1)···N(4) 88.65. H atoms that are not involved in intramolecular bonding have been omitted for clarity. Colour: bond = Blue: Hydrogen bond (X/Se···H), Yellow: Halogen Bond (Se···X and X···X), Red: π-interactions (Se···C), Pink: Chalcogen Bond (Br···O).

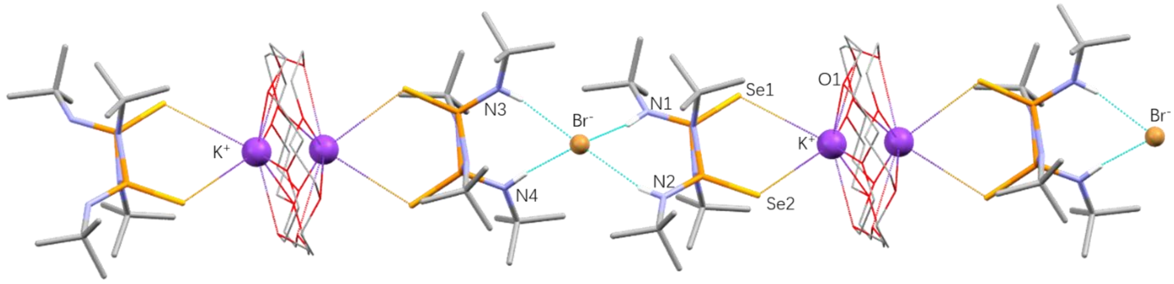

Figure S23: Crystal 2 of (**$Se_2P_2N_2$**)·(**18-crown-6 ether**)·(**KBr**) Selected lengths of intermolecular interactions [Å] and Angles [°]: Se(1)···K^+ 3.755, Se(2)··· K^+ 3.742, N(1)···Br^- 3.430, N(2)···Br^- 3.361, N(3)···Br^- 3.430, N(4)···Br^- 3.361; N(1)···Br^-···N(2) 76.13, N(3)···Br^-···N(4) 76.13, Se(1)···K^+···Se(2) 82.34, O(1)···K^+ 2.930. H atoms that are not involved in intramolecular bonding have been omitted for clarity.

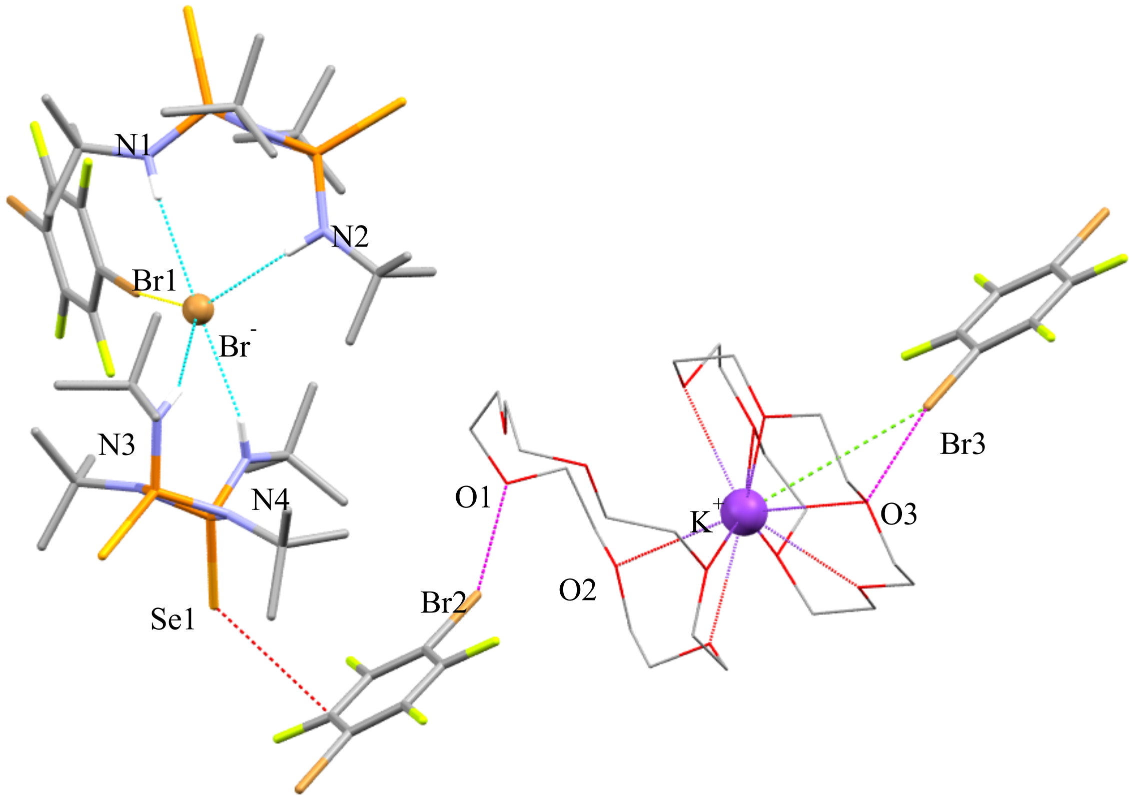

Figure S24: Crystal 3 (**$Se_2P_2N_2$**)·(**18-crown-6 ether**)·(**KBr**))·(**DBTFB**); Selected lengths of intermolecular interactions [Å] and Angles [°]: N(1)···Br^- 3.463, N(2)···Br^- 3.559, N(3)···Br^- 3.510, N(4)···Br^- 3.430, Br(1)···Br^- 3.573, Se(1)···C(1) 3.479, Br(2)···O(1) 2.995, O(2)···K^+ 3.118, K^+···O(3) 3.104, O(3)···Br(3) 2.937, K^+···**Br(3)** 4.418; N(1)···Br^-···N(2) 71.91, N(3)···Br^-···N(4) 72.56. H atoms that are not involved in intramolecular bonding have been omitted for clarity. Colour: bond = Blue: Hydrogen bond (X/Se···H), Green: Cation-Dipole interaction (K^+···Br),c Yellow: Halogen Bond (Se···X and X···X), Red: π-interactions (Se···C), Pink: Chalcogen Bond (Br···O).

5. Expanded Manuscript Figures

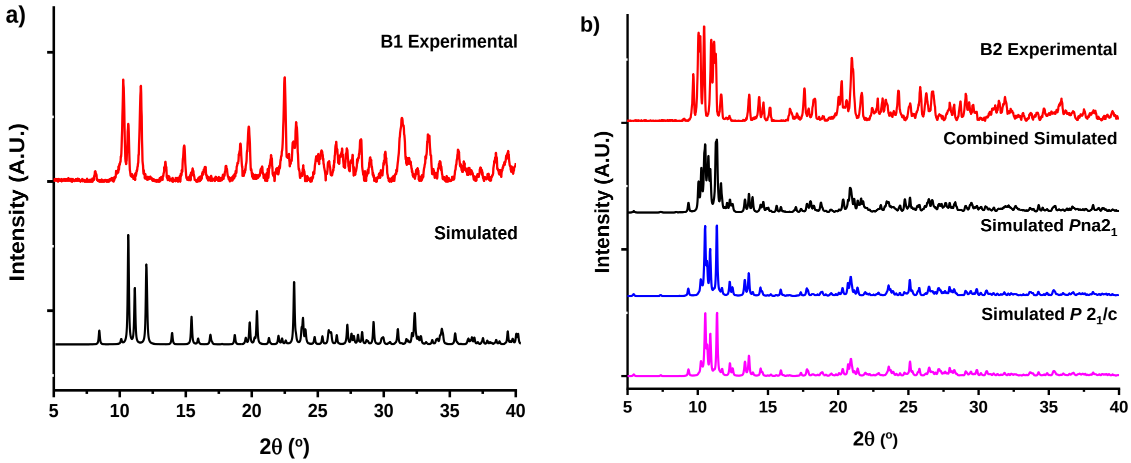

Figure 1. Overlay of powder diffraction patterns of the mechanochemically obtained powders and simulated patterns from single crystal data of the binary cocrystals of **$Se_2P_2N_2$** and a) **DBTFB**, b) **DMU**; two polymorphs of the binary cocrystal were obtained. For expanded view see SI

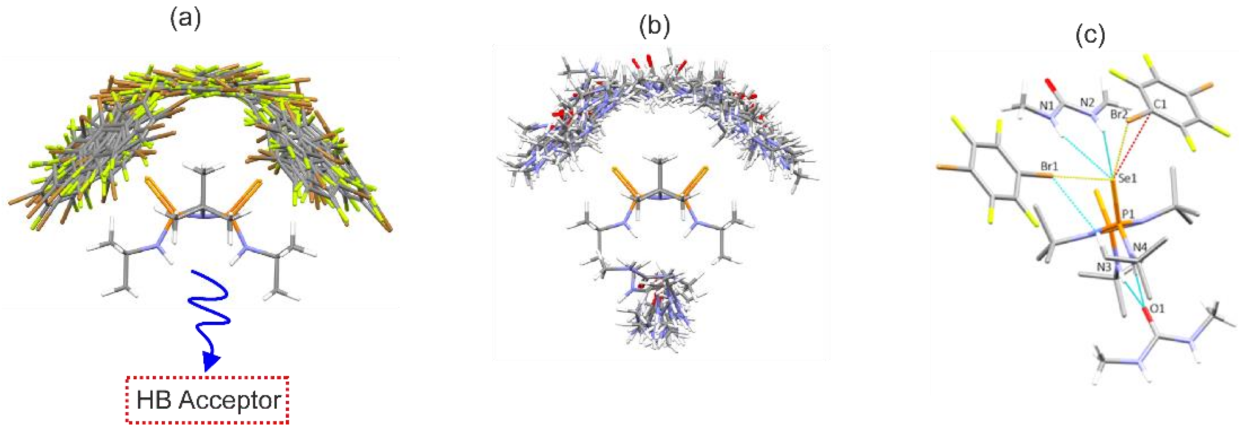

Figure 2. Monte Carlo simulated trajectories for the 100 most stable binding geometries between **$Se_2P_2N_2^{exo,exo}$** and either (a) **DBTFB** or (b) **DMU**. (c) Fragment of the crystal structure of the ternary cocrystal **T1** depicting the Se···Br, Se···H-N and Se···π interactions. Some C-H hydrogens are omitted for clarity.

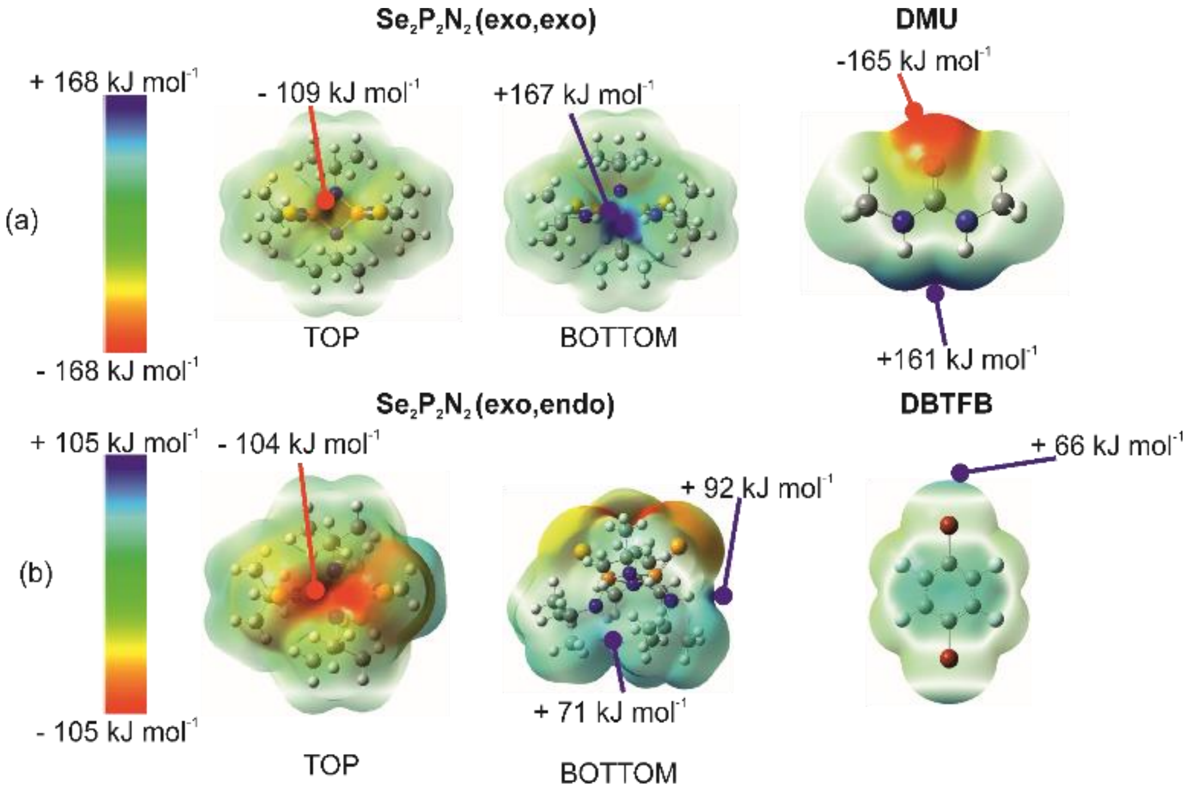

Figure 3. Molecular electrostatic potential (MEP) surfaces for (a) **$Se_2P_2N_2$** (top and bottom views) and **DMU**; (b) **$Se_2P_2N_2$** (top and bottom views) and **DBTFB**. See SI for expanded version.

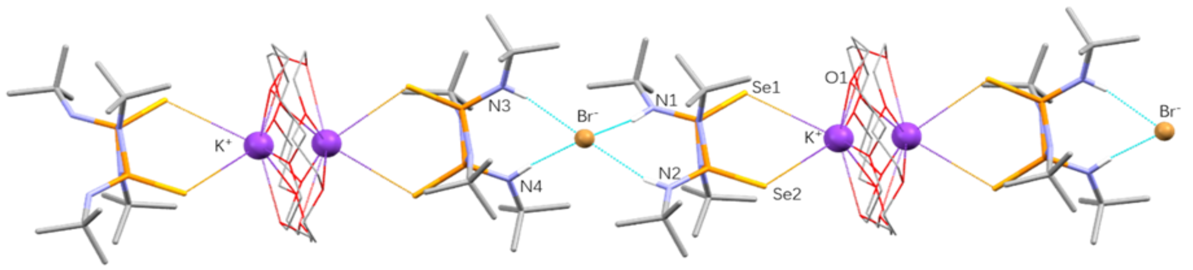

Expanded Figure 4. Fragment of the crystal structure of the ternary ICC, (**$Se_2P_2N_2$**)·(**crown**)·(**KBr**) **T2**, forming one-dimensional chains linked by Se···K^+ and Br^-···H-N interactions. The crown and K^+ are positionally disordered over two sites. Some C-H hydrogens are omitted for clarity. For expanded version see SI.

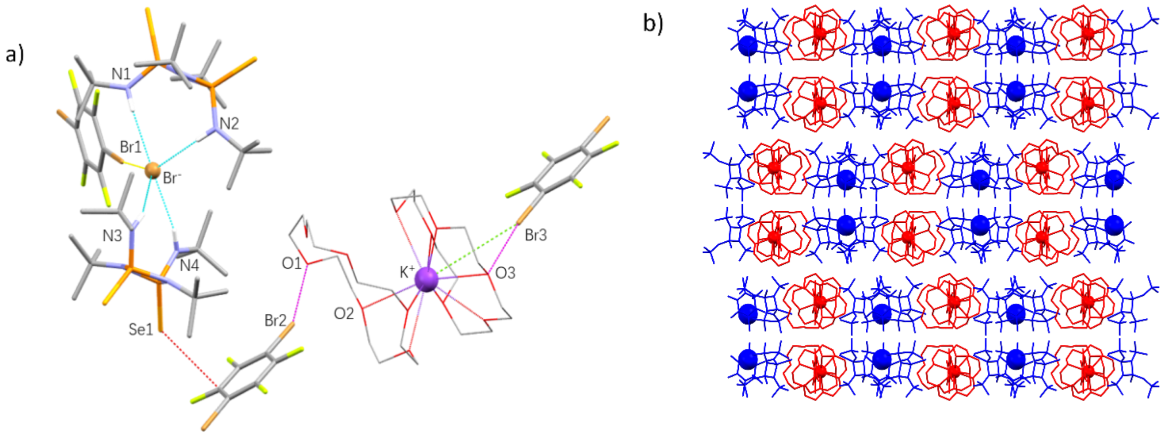

Expanded Figure 5. a) Fragment of the crystal structure of the four-component ICC, **($Se_2P_2N_2$)$_2$•(DBTFB)$_2$•(crown)$_2$•(KBr)** (**Q**), depicting the N-H···Br⁻, O···Br, Br···Br⁻, O···K⁺ and Se···π interactions; b) Fragment of the crystal structure of the four-component ICC, **Q**, depicting the packing motif as viewed down the crystallographic c-axis. The red and blue domains are linked by Se···π interactions. The blue spheres are the Br⁻ anions and the red spheres are the K⁺ cations. For expanded version see SI.

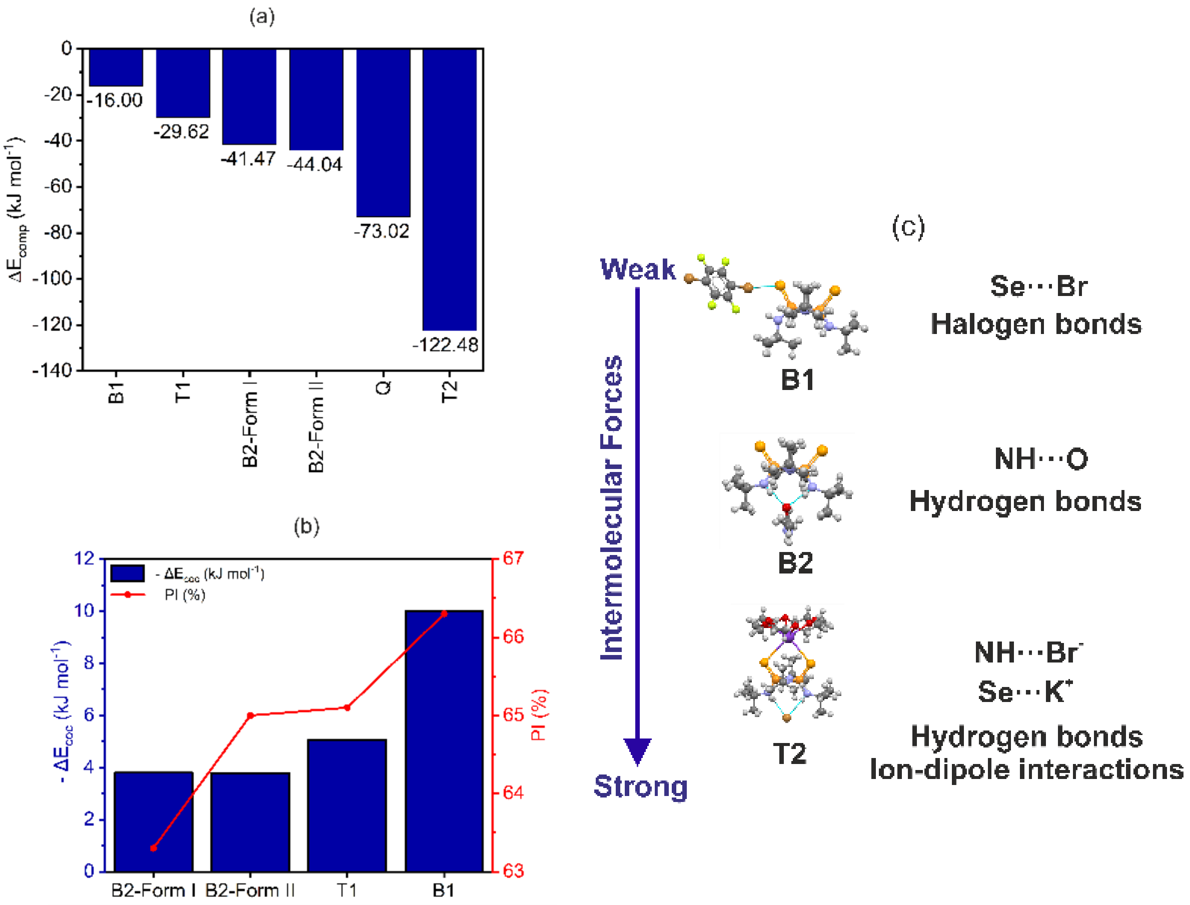

Expanded Figure 6: (a) Bar graph of the trend in the complexation energy (ΔE_{comp}) for each cocrystal. (b) Variation in the DFT energy for the formation of the molecular cocrystals ($-\Delta E_{coc}$) **B1**, **B2** and **T1** with the packing index (PI) of each cocrystal. (c) Illustration of the observed synthons and relative strengths of the intermolecular interactions observed in the various cocrystals. For expanded version see SI.

6. References

[1] Kresse, G.; Furthmüller, J., Efficiency of ab-initio total energy calculations for metals and semiconductors using a plane-wave basis set. *Computational Materials Science* **1996,** *6* (1), 15-50.

[2] Kresse, G.; Furthmüller, J., Efficient iterative schemes for ab initio total-energy calculations using a plane-wave basis set. *Physical Review B* **1996,** *54* (16), 11169-11186.

[3] Kresse, G.; Hafner, J., Ab initio molecular dynamics for liquid metals. *Physical Review B* **1993,** *47* (1), 558-561.

[4] Kresse, G.; Joubert, D., From ultrasoft pseudopotentials to the projector augmented-wave method. *Physical Review B* **1999,** *59* (3), 1758-1775.

[5] Perdew, J. P.; Burke, K.; Ernzerhof, M., Generalized gradient approximation made simple. *Phys. Rev. Lett.* **1996,** *77* (18), 3865-3868.

[6] Grimme, S.; Antony, J.; Ehrlich, S.; Krieg, H., A consistent and accurate ab initio parametrization of density functional dispersion correction (DFT-D) for the 94 elements H-Pu. *J. Chem. Phys.* **2010,** *132* (15), 154104.

[7] Björkman, T., CIF2Cell: Generating geometries for electronic structure programs. *Computer Physics Communications* **2011,** *182* (5), 1183-1186.

[8] Frisch, M. J.; Trucks, G. W.; Schlegel, H. B.; Scuseria, G. E.; Robb, M. A.; Cheeseman, J. R.; Scalmani, G.; Barone, V.; Mennucci, B.; Petersson, G. A.; Nakatsuji, H.; Caricato, M.; Li, X.; Hratchian, H. P.; Izmaylov, A. F.; Bloino, J.; Zheng, G.; Sonnenberg, J. L.; Hada, M.; Ehara, M.; Toyota, K.; Fukuda, R.; Hasegawa, J.; Ishida, M.; Nakajima, T.; Honda, Y.; Kitao, O.; Nakai, H.; Vreven, T.; Montgomery Jr., J. A.; Peralta, J. E.; Ogliaro, F.; Bearpark, M. J.; Heyd, J.; Brothers, E. N.; Kudin, K. N.; Staroverov, V. N.; Kobayashi, R.; Normand, J.; Raghavachari, K.; Rendell, A. P.; Burant, J. C.; Iyengar, S. S.; Tomasi, J.; Cossi, M.; Rega, N.; Millam, N. J.; Klene, M.; Knox, J. E.; Cross, J. B.; Bakken, V.; Adamo, C.; Jaramillo, J.; Gomperts, R.; Stratmann, R. E.; Yazyev, O.; Austin, A. J.; Cammi, R.; Pomelli, C.; Ochterski, J. W.; Martin, R. L.; Morokuma, K.; Zakrzewski, V. G.; Voth, G. A.; Salvador, P.; Dannenberg, J. J.; Dapprich, S.; Daniels, A. D.; Farkas, Ö.; Foresman, J. B.; Ortiz, J. V.; Cioslowski, J.; Fox, D. J. Revision D.01; Gaussian, Inc.: Wallingford, CT, USA, 2009.

[9] Boys, S. F.; Bernardi, F., The calculation of small molecular interactions by the differences of separate total energies. Some procedures with reduced errors. *Mol. Phys.* **2002,** *100* (1), 65-73.

[10] Dennington, R.; Keith, T.; Millam, J. *GaussView*, 5.0.9; Semichem Inc., Shawnee Mission KS: 2009.

[11] Besler, B. H.; Merz Jr, K. M.; Kollman, P. A., Atomic charges derived from semiempirical methods. *J. Comput. Chem.* **1990,** *11* (4), 431-439.

[12] Singh, U. C.; Kollman, P. A., An approach to computing electrostatic charges for molecules. *J. Comput. Chem.* **1984,** *5* (2), 129-145.

[13] *Dassault Systèmes BIOVIA*, Version 8.0.100.21; San Diego: Dassault Systèmes, 2014.

[14] Mayo, S. L.; Olafson, B. D.; Goddard, W. A., DREIDING: a generic force field for molecular simulations. *J. Phys. Chem.* **1990,** *94* (26), 8897-8909.

[15] Rappe, A. K.; Goddard, W. A., Charge equilibration for molecular dynamics simulations. *J. Phys. Chem.* **1991,** *95* (8), 3358-3363.
